# CUL5 E3 ubiquitin ligase regulates the evasion of bladder cancer cells to CD8^+^ T cell-mediated killing by inhibiting autophagy

**DOI:** 10.1371/journal.pbio.3003647

**Published:** 2026-02-09

**Authors:** Xincheng Gao, Yanchao Yu, Jiayin Sun, Huayuan Zhao, Yongqiang You, Xin Shi, Kang Wang, Sijia Hong, Xing Xiong, Chao Huang, Hui Zhang, Guosong Jiang

**Affiliations:** 1 Department of Urology, Union Hospital, Tongji Medical College, Huazhong University of Science and Technology, Wuhan, China; 2 Department of Urology, Zhongnan Hospital of Wuhan University, Wuhan, China; 3 Institute of Urology, The Affiliated Luohu Hospital of Shenzhen University, Shenzhen University, Shenzhen, China; University of Bern, SWITZERLAND

## Abstract

CD8^+^ T cells are capable of specifically targeting and eliminating malignant tumor cells, but tumor cells can develop resistance mechanisms to escape CD8^+^ T cell-mediated killing. Here, we performed a whole genome CRISPR-Cas9 knockout screen under CD8^+^ T cells pressure and identified the E3 ubiquitin ligase CUL5 as an essential factor required for escaping CD8^+^ T cells killing in bladder cancer cells. We found that CUL5 knockout promoted the sensitivity of bladder cancer cells to CD8^+^ T cell-mediated killing both in vivo and in vitro. Mechanistically, CUL5 loss reduced the ubiquitination of PTBP1, which regulated alternative splicing of RUBCN pre-mRNA and led to an increase in the levels of the RUBCN-S isoform, thereby preventing immune evasion of bladder cancer cells by inhibiting autophagy. Importantly, CUL5 knockout significantly enhanced the efficacy of anti-PD-1 immunotherapy in a xenograft model. Collectively, these findings reveal a novel mechanism of bladder cancer immune evasion, providing potential targets for cancer immunotherapy.

## Abstract 2

CD8+ T cells can specifically target and eliminate malignant tumor cells, but tumors are known to develop resistance mechanisms to escape CD8+ T-cell-mediated killing. This study shows that loss of the E3 ubiquitin ligase CUL5 enhances CD8+ T cell cytotoxicity and anti PD-1 efficacy by inhibiting autophagy.

## Introduction

Bladder cancer is one of the most common malignant tumors worldwide, and the incidence and mortality of bladder cancer are increasing every year [1,2]. Despite the clinical success of immune checkpoint blockade (ICB) therapies, the majority of patients do not respond or ultimately relapse due to tumor-acquired resistance [3]. The resistance mechanisms operate in part through tumor-intrinsic resistance to killing mediated by CD8^+^ T cells [4]. CD8^+^ T cells are key effectors of tumor immunity on the basis of their ability to specifically recognize and kill tumor cells [5,6]. However, cancer cells exploit multiple mechanisms to achieve immune evasion [7,8]. A small number of genes, such as PD-L1 [9], that enable tumors to evade the immune system have been discovered and are the focus of clinical development efforts [3,10]. Nevertheless, the more genes that mediate resistance to the killing by CD8^+^ T cells in bladder cancer cells are unclear. Therefore, there is an urgent need to identify the mechanisms by which bladder cancer cells resist CD8^+^ T cell-mediated killing.

Ubiquitination is a common posttranslational modification of proteins that regulates diverse biological processes [11,12]. The dysfunction of E3 ubiquitin ligases is a pivotal regulatory factor involved in the development, progression and therapeutic response of various cancers [13]. In addition, the ubiquitin system also plays an important function in anti-tumor immunity. For instance, E3 ubiquitin ligase RNF31 ablation sensitized tumor cells, including melanoma, breast and colorectal cancers, to CD8^+^ T cells killing [14]. Notably, Cullin5 (CUL5), a member of the cullin protein family, is a key scaffold molecule in the cullin-ring E3 ubiquitin ligase complex [15]. Recently, CUL5 acts as a negative regulator of the central signaling pathway in CD8^+^ T cells, and loss of CUL5 in mouse CD8^+^ T cells markedly enhances their primary tumor growth inhibitory ability [16]. Furthermore, it is reported that a CUL5-based complex plays an essential role in viral immune evasion by regulating the stability of SARS-CoV-2 ORF9b protein [17]. However, the function of CUL5 in tumor cells to regulate CD8^+^ T cell-mediated killing has not been revealed, and the role of CUL5 in bladder cancer immune evasion still needs to be further explored.

In recent years, the introduction of functional genetic CRISPR screening technology has dramatically enhanced our ability to understand genes involved in tumor immune evasion and resistance to ICB therapies [5,18–20]. To identify cancer-intrinsic genes that regulate sensitivity to CD8^+^ T cell-mediated killing, we utilized a whole-genome CRISPR-Cas9 screen in bladder cancer cell lines that were co-cultured with CD8^+^ T cells. We identified CUL5 as a target that decreased the sensitivity of bladder cancer cells to CD8^+^ T cell-mediated killing. More specifically, knockout of CUL5 could regulate the alternative splicing (AS) of RUBCN pre-mRNA via PTBP1, resulting in an increase in the levels of the RUBCN-S isoform, and subsequently inhibiting autophagy to prevent immune evasion in bladder cancer cells. Together, these studies demonstrated the role of CUL5 in immune evasion of bladder cancer and provided a potential therapeutic target for improving the efficacy of bladder cancer immunotherapy.

## Results

### CUL5 knockout increases the sensitivity of bladder cancer cells to CD8^+^ T cell-mediated killing

**Fig 1:**
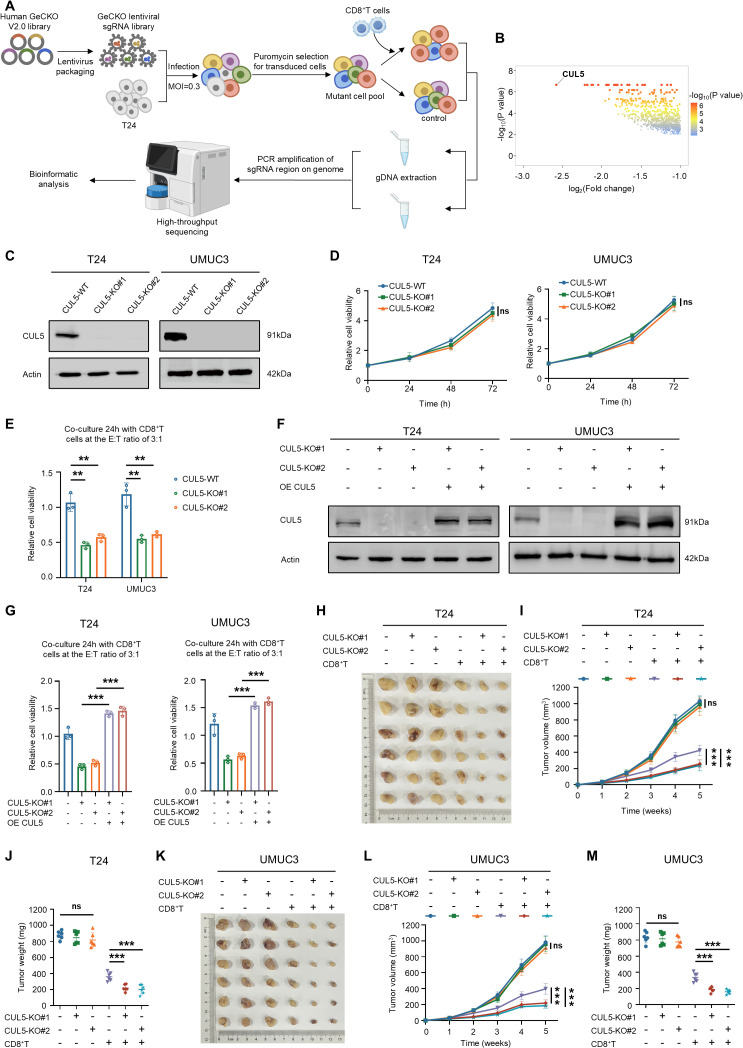
CUL5 knockout increases the sensitivity of bladder cancer cells to CD8^+^ T cell-mediated killing. **(A)** Schematic of CRISPR-Cas9 screening strategy (The cartoon was created in BioRender. Naiwei, Z. (2025) https://BioRender.com/qut2r9n). **(B)** Volcano plots showing results of genome-wide CRISPR-Cas9 negative screen (*P* < 0.01, log_2_Fold change < −1.0, and good sgRNAs ≥ 5). **(C)** The efficiency of CUL5-KO in T24 and UMUC3 cells was detected by western blotting. **(D)** CCK-8 assay revealed the cell viability of CUL5-WT, CUL5-KO#1, and CUL5-KO#2 bladder cancer cells at the indicated time points. **(E)** CUL5-WT, CUL5-KO#1 and CUL5-KO#2 bladder cancer cells were co-cultured with CD8^+^ T cells for 24 h, and cell viability was measured by CCK-8. **(F)** The expression levels of CUL5 in T24 and UMUC3 cells were detected by western blotting. **(G)** The different groups of bladder cancer cells were co-cultured with CD8^+^T cells for 24 h, and cell viability was measured by CCK-8. **(H–J)** One week after CUL5-WT, CUL5-KO#1, and CUL5-KO#2 T24 cells were subcutaneously injected into the right flanks of nude mice, the experimental group was injected with activated CD8^+^T cells via the tail vein (*n* = 6 for each group). Representative images of xenograft tumors were shown in (H), and growth curves (I) and the tumors weight for each group were measured (J). **(K–M)** One week after CUL5-WT, CUL5-KO#1, and CUL5-KO#2 UMUC3 cells were subcutaneously injected into the right flanks of nude mice, the experimental group was injected with activated CD8^+^ T cells via the tail vein (*n* = 6 for each group). Representative images of xenograft tumors were shown in (K), and growth curves (L), and the tumors weight for each group were measured (M). Data are presented as the means ± SD from three independent experiments. ANOVA was applied to analyze and compare the data in D, I and L. Student *t* test was applied to analyze and compare the data in E, G, J, and M. ns, nonsignificant; **P < 0.01; ***P < 0.001. The raw data underlying all figures can be found in S1 Data. Original blots can be found in S1 Raw Images.

To uncover tumor-intrinsic genes that regulate sensitivity to CD8^+^ T cell-mediated killing, we performed genome-wide CRISPR/Cas9 knockout library screen in T24 cells to test their perturbation effects when co-cultured with CD8^+^ T cells (Fig 1A). From this CRISPR/Cas9 knockout library screen, sgRNAs targeting 1002 genes were negatively selected (*P* < 0.01, log_2_Fold change < −1.0, and good sgRNAs ≥ 5) in T24 cells challenged by CD8^+^ T cells compared to the unchallenged control (S1 Table). Among the list of genes, the sgRNA for the E3 ubiquitin ligase CUL5 was the most significantly depleted (Fig 1B and S1 Table), which indicated that targeting the CUL5 in bladder cancer cells has the potential to increase their sensitivity to CD8^+^ T cell-mediated killing.

To verify the role of CUL5 in bladder cancer cells, we deleted the CUL5 gene in T24 and UMUC3 cells by CRISPR/Cas9 gene editing (Fig 1C). Knockout of CUL5 alone did not significantly impair the viability of bladder cancer cells (Fig 1D). However, CUL5-KO bladder cancer cells exhibited increased sensitivity to CD8^+^ T cell-mediated killing (Fig 1E). Next, we overexpressed CUL5 in bladder cancer cells where CUL5 had been previously knocked out (Fig 1F). Rescuing CUL5 expression restored resistance to CD8^+^ T cell-mediated killing (Fig 1G). Subsequently, we established a bladder cancer xenograft mouse model to assess whether CUL5-KO has a similar impact in vivo. CD8^+^ T cell-treated mice bearing CUL5-KO tumors showed a significant inhibition of tumor growth, whereas untreated mice with CUL5-KO tumors showed little effect on tumor growth (Fig 1H–1M). Overall, these results suggested that the CUL5 knockout significantly increased the sensitivity of bladder cancer cells to CD8^+^ T cell-mediated killing both in vivo and in vitro.

### CUL5 interacts with PTBP1 protein

**Fig 2:**
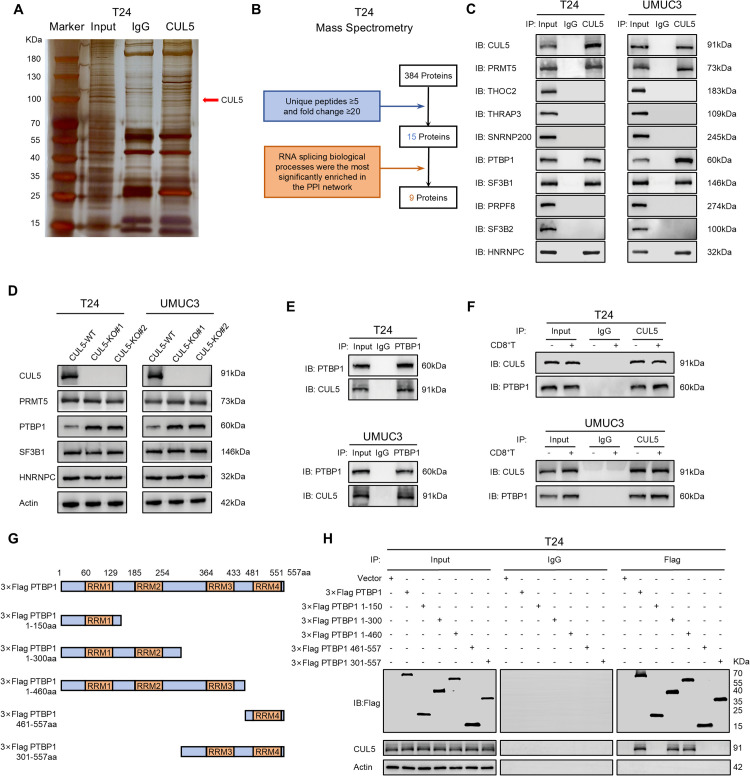
CUL5 interacts with PTBP1 protein. **(A)** Silver staining showed the proteins pulled down by CUL5 from the lysates of T24 cells. **(B)** Analysis pipeline was performed to identify potential proteins that interact with CUL5: **(i)** The proteins with unique peptides ≥5 and fold change ≥20 in T24-MS were selected; **(ii)** The RNA splicing biological process exhibited the most significant enrichment within the protein-protein interaction (PPI) network predicted by the STRING database (https://cn.string-db.org/). **(C)** Co-IP assay was performed using an antibody specific for CUL5 to immunoprecipitate proteins from lysates of T24 and UMUC3 cells. The precipitate was subjected to western blotting with the antibodies against CUL5, PRMT5, THOC2, THRAP3, SNRNP200, PTBP1, SF3B1, PRPF8, SF3B2, and HNRNPC. **(D)** Western blotting with the indicated antibodies in T24 and UMUC3 cells following CUL5 knockout. **(E)** Co-IP assay using antibody specific for PTBP1 showed the interaction between CUL5 and PTBP1 in T24 and UMUC3 cells. The precipitate was subjected to western blotting with the antibodies against PTBP1 and CUL5. **(F)** Co-IP assay using antibody specific for CUL5 showed that the interaction between CUL5 and PTBP1 was not affected when bladder cancer cells were co-cultured with CD8^+^ T cells. The precipitate was subjected to western blotting with the antibodies against CUL5 and PTBP1. **(G)** Schematic diagram revealed the domains of PTBP1 full-length or truncations. **(H)** Co-IP assay using antibody specific for Flag showed the interaction between CUL5 and full-length or truncations of Flag-tagged recombinant PTBP1 in T24 cells. The precipitate was subjected to western blotting with the antibodies against Flag, CUL5, and β-actin. Original blots can be found in S1 Raw Images.

Given that CUL5 is a key component of Cullin-RING E3 ubiquitin ligase 5 (CRL5), we performed mass spectrometry to identify potential proteins that interact with CUL5 (Fig 2A and S3 Table). Following the analysis pipeline, there were nine proteins mainly enriched in RNA splicing biological process (Figs 2B, S1A, and S1B). We found that PRMT5, PTBP1, SF3B1 and HNRNPC may be potential substrates of CUL5 by Co-IP assay (Fig 2C). Further analysis of their expression in CUL5-KO bladder cancer cells revealed that the protein level of PTBP1 was significantly increased upon CUL5-KO (Fig 2D). Therefore, we hypothesized that PTBP1 was most likely to be a target protein regulated by CUL5. Afterward, our Co-IP assay confirmed the interaction between CUL5 and PTBP1 (Fig 2E). In addition, the interaction between CUL5 and PTBP1 was not affected when bladder cancer cells were co-cultured with CD8^+^ T cells (Fig 2F). These results demonstrated that PTBP1 acted as a substrate protein of CUL5 in bladder cancer.

PTBP1 consists of 557 amino acids, featuring four RNA recognition motif (RRM) domains that not only bind to RNA but also serve as interfaces for protein–protein interactions [21–23]. To clarify which domain of PTBP1 contributed to the interaction with CUL5, we constructed five truncated mutants of PTBP1 (Fig 2G). The PTBP1-RRM2 domain was identified as crucial for its interaction with CUL5 (Fig 2H). The above experimental data confirmed that CUL5 interacted with the PTBP1-RRM2 domain.

### CUL5 mediates the K48-linked polyubiquitination of PTBP1

**Fig 3:**
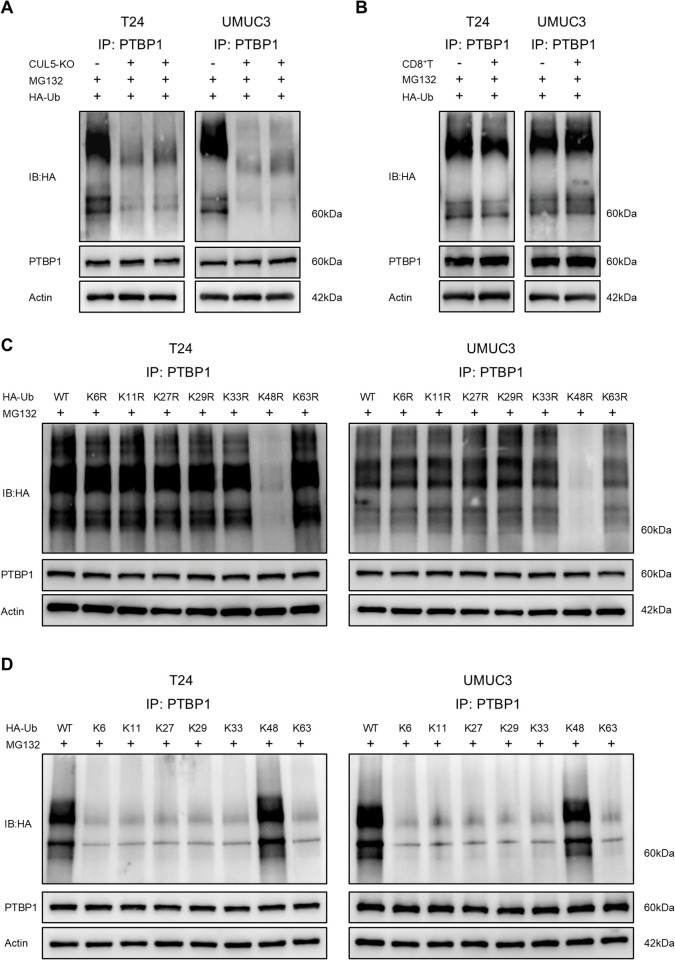
CUL5 mediates the K48-linked polyubiquitination of PTBP1. **(A)** Western blot analysis of PTBP1 ubiquitination in CUL5-WT, CUL5-KO#1, and CUL5-KO#2 bladder cancer cells transfected with HA-Ub. Cells were treated with 10 μM MG132 for 24 h. **(B)** Western blotting was used to detect ubiquitination of PTBP1 in CUL5-WT bladder cancer cells co-cultured with CD8^+^ T cells. Cells were treated with 10 μM MG132 for 24 h. **(C)** PTBP1 poly-ubiquitination linkage was examined by transfecting HA-tagged WT or indicated ubiquitin mutants containing only Lys 6/11/27/29/33/48/63 mutated to arginine into T24 and UMUC3 cells, followed by WB analysis of HA-Ub in anti-PTBP1 IP products. Cells were treated with 10 μM MG132 for 24 h. **(D)** PTBP1 poly-ubiquitination linkage was examined by transfecting HA-tagged WT or indicated ubiquitin mutants (K6/K11/K27/K29/K33/K48/K63) into T24 and UMUC3 cells, followed by WB analysis of HA-Ub in anti-PTBP1 IP products. Cells were treated with 10 μM MG132 for 24 h. Original blots can be found in S1 Raw Images.

We further investigated the effect of CUL5 on PTBP1 polyubiquitination. In the presence of the proteasome inhibitor MG132, CUL5-KO significantly inhibited PTBP1 polyubiquitination (Fig 3A). Moreover, the polyubiquitination levels of PTBP1 were not altered after co-culturing CUL5-WT bladder cancer cells with CD8^+^ T cells (Fig 3B). Ubiquitination through seven types of linkages between ubiquitin molecules, including Lys6, Lys11, Lys27, Lys29, Lys33, Lys48, and Lys63 [24]. Consequently, we mutated each of the lysines (e.g., K6R-Ub) or six of the seven lysines to arginine (e.g., K6-Ub) in the ubiquitin plasmids, and demonstrated that K48R-Ub inhibited the accumulation of polyubiquitinated PTBP1 (Fig 3C), whereas K48-Ub could be linked to PTBP1 as efficiently as wild-type ubiquitin (Fig 3D). These results indicated that CUL5 induced K48-linked polyubiquitination of PTBP1.

### Loss of CUL5 regulates RUBCN alternative splicing through PTBP1

**Fig 4:**
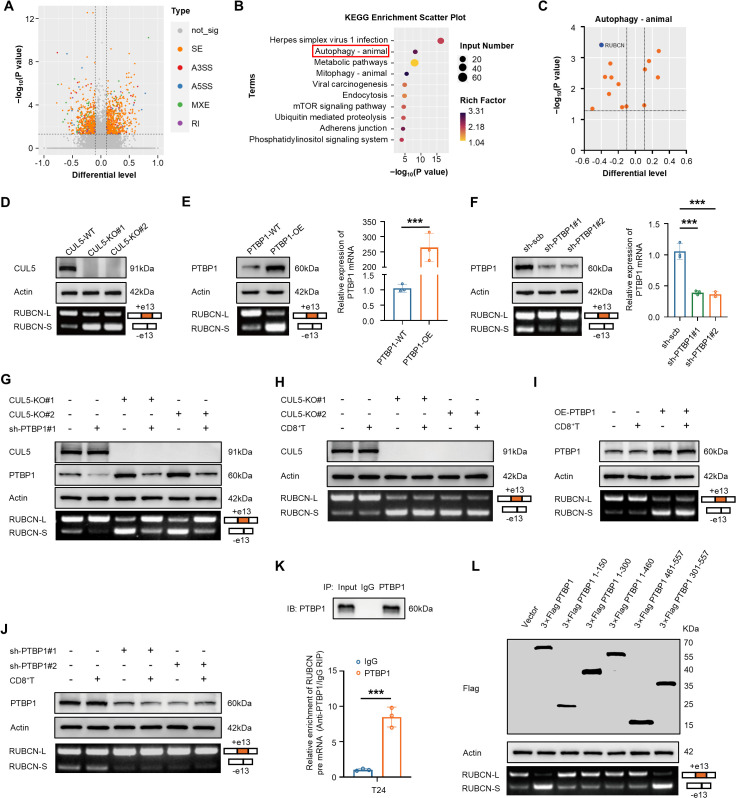
Loss of CUL5 regulates RUBCN alternative splicing by PTBP1. **(A)** Volcano plot of differential alternative splicing (AS) events screening in CUL5-KO compared with CUL5-WT T24 cells. The AS events are classified into 5 types: skipped exon (SE), alternative 3′ splice site (A3SS), alternative 5′ splice site (A5SS), mutually exclusive exon (MXE), and retained intron (RI). **(B)** KEGG enrichment analysis revealed the enriched pathways for differential AS genes upon CUL5 knockout in T24 cells. **(C)** The volcano plot showed that RUBCN had the most significant changes in the SE category of autophagy pathways. **(D)** Agarose gel electrophoresis analysis of the RUBCN isoforms in CUL5-KO T24 cells. The structure of each PCR product was indicated schematically on the right, and the alternative exon affected by CUL5 was painted in orange. **(E)** Agarose gel electrophoresis analysis of the RUBCN isoforms in PTBP1-overexpressed T24 cells. The efficiency of PTBP1-overexpressed in T24 cells was detected by western blotting (left) and qRT-PCR (right). **(F)** Agarose gel electrophoresis analysis of the RUBCN isoforms in PTBP1-knockdown T24 cells. The efficiency of PTBP1-knockdown in T24 cells was detected by western blotting (left) and qRT-PCR (right). **(G)** Western blotting with the indicated antibodies in CUL5-WT and CUL5-KO T24 cells transfected with scramble or sh-PTBP1#1, and agarose gel electrophoresis for analysis of RUBCN isoforms. **(H)** Agarose gel electrophoresis analysis of the RUBCN isoforms in CUL5-KO T24 cells co-cultured with CD8^+^ T cells. **(I)** Agarose gel electrophoresis analysis of the RUBCN isoforms in PTBP1-overexpressed T24 cells co-cultured with CD8^+^ T cells. **(J)** Agarose gel electrophoresis analysis of the RUBCN isoforms in PTBP1-knockdown T24 cells co-cultured with CD8^+^ T cells. **(K)** RIP assays in T24 cells using PTBP1 and IgG antibody. The precipitate was subjected to western blotting with the antibody against PTBP1. The PTBP1-enriched RUBCN pre mRNA relative to the IgG-enriched value was calculated by qRT-PCR. **(L)** Agarose gel electrophoresis analysis of the RUBCN isoforms in T24 cells transfected with vector, full-length or truncations of Flag-tagged recombinant PTBP1. Data are presented as the means ± SD from three independent experiments. Student *t* test was applied to analyze and compare the data in E, F and **K.** ***P < 0.001. The raw data underlying all figures can be found in S1 Data. Original blots and gels can be found in S1 Raw Images.

Considering that PTBP1 acts as a classical splicing factor [25], we conducted high-throughput sequencing of RNA (RNA-Seq) on the CUL5-WT and CUL5-KO T24 cells to analyze the changes of AS. Among the various AS events detected, 71.3% belonged to the skipped exon (SE) category (Fig 4A and S4 Table). We found that the genes were mainly involved in autophagy pathway (Fig 4B), with the autophagy repressor RUBCN being the most significantly altered in SE category (Fig 4C). Based on the two isoforms of RUBCN identified through RNA-seq analysis, we designed primers spanning RUBCN exon 13 to amplify both the exon 13-skipping and exon 13-including isoforms (S2 Table). Consistent with the RNA-Seq results, CUL5-KO significantly resulted in skipping of RUBCN exon 13, specifically manifesting as increased expression of the short RUBCN (RUBCN-S, skipping exon 13) isoform and decreased expression of the long RUBCN (RUBCN-L, including exon 13) isoform (Figs 4D and S2A). Similarly, overexpression of PTBP1 in CUL5-WT bladder cancer cells resulted in skipping of RUBCN exon 13 (Figs 4E and S2B). Conversely, knockdown of PTBP1 inhibited the skipping of RUBCN exon 13 and reversed the skipping of RUBCN exon 13 that was caused by CUL5 knockout (Figs 4F, 4G, S2C, and S2D), which demonstrated that CUL5 deletion regulated AS of RUBCN via PTBP1. We observed that the expressions of RUBCN-L and RUBCN-S remained unchanged in bladder cancer cells co-cultured with CD8^+^ T cells (Figs 4H–4J and S2E–S2G). In addition, the interaction between PTBP1 and RUBCsN pre-mRNA was validated through RIP assay (Figs 4K and S2H). The introduction of domain deletion mutants of PTBP1 (PTBP1-ΔRRM2-4, -ΔRRM3-4, -ΔRRM4, -ΔRRM1-3) failed to result in RUBCN splicing, whereas the deletion mutant PTBP1-ΔRRM1–2 could lead to the splicing of RUBCN, suggesting that the domains RRM3-4 of PTBP1 are required for efficient splicing of RUBCN pre-mRNA (Figs 4L and S2I). Collectively, these results showed that CUL5 deletion regulated AS of RUBCN pre-mRNA by PTBP1 RRM3-4 domains.

### Rubicon-S inhibits autophagy in bladder cancer cells but Rubicon-L does not

**Fig 5:**
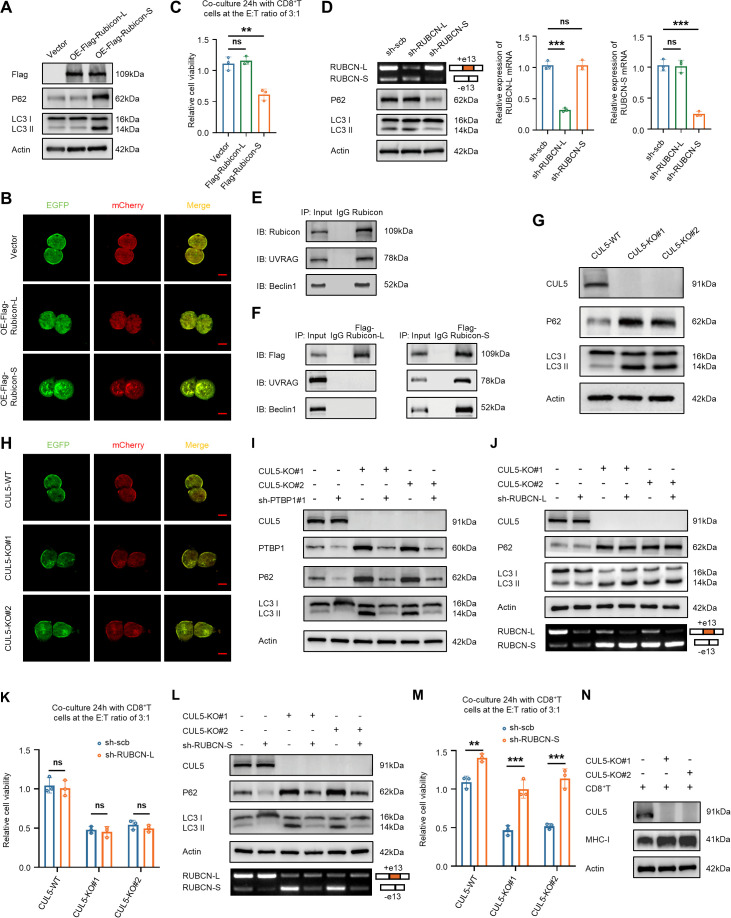
CUL5 knockout increases the sensitivity of bladder cancer cells to CD8^+^ T cell-mediated killing by inhibiting autophagy. **(A)** The expression levels of LC3B and P62 in T24 cells transfected with vector, Flag-Rubicon-L, or Flag-Rubicon-S were detected by western blotting. **(B)** T24 cells were transfected with vector, Flag-Rubicon-L or Flag-Rubicon-S plasmids, and those co-transfected with mCherry-EGFP-LC3B. The autophagosomes with yellow puncta and autolysosomes with red puncta. Bar: 10 μm. **(C)** T24 cells were transfected with the vector, Flag-Rubicon-L, and Flag-Rubicon-S plasmids, and then co-cultured with CD8^+^ T cells, and cell viability was measured by CCK-8. **(D)** Western blotting with the indicated antibodies in T24 cells transfected with scramble, sh-RUBCN-L or sh-RUBCN-S. The efficiency of RUBCN-L knockdown and RUBCN-S knockdown in T24 cells was detected by agarose gel electrophoresis (left) and qRT-PCR (right). **(E)** Co-IP assay using antibody specific for Rubicon showed that Rubicon interacted with UVRAG and Beclin1 in T24 cells. The precipitate was subjected to western blotting with the antibodies against Rubicon, UVRAG, and Beclin1. **(F)** Co-IP assay using antibody specific for Flag showed that Flag-Rubicon-S interacted with UVRAG and Beclin1(Right), while Flag-Rubicon-L could not bind to UVRAG and Beclin1 in T24 cells (Left). The precipitate was subjected to western blotting with the antibodies against Flag, UVRAG, and Beclin1. **(G)** The expression levels of LC3B and P62 in CUL5-KO T24 cells were detected by western blotting. **(H)** CUL5-WT, CUL5-KO#1, and CUL5-KO#2 T24 cells were transfected with the mCherry-EGFP-LC3B. The autophagosomes with yellow puncta and autolysosomes with red puncta. Bar: 10 μm. **(I)** Western blotting with the indicated antibodies in CUL5-WT and CUL5-KO T24 cells transfected with scramble or sh-PTBP1#1. **(J)** Western blotting with the indicated antibodies in CUL5-WT and CUL5-KO T24 cells transfected with scramble or sh-RUBCN-L, and agarose gel electrophoresis for analysis of RUBCN isoforms. **(K)** CUL5-WT, CUL5-KO#1, and CUL5-KO#2 T24 cells were transfected with the scramble or sh-RUBCN-L, and then co-cultured with CD8^+^ T cells, and cell viability was measured by CCK-8. **(L)** Western blotting with the indicated antibodies in CUL5-WT and CUL5-KO T24 cells transfected with scramble or sh-RUBCN-S, and agarose gel electrophoresis for analysis of RUBCN isoforms. **(M)** CUL5-WT, CUL5-KO#1, and CUL5-KO#2 T24 cells were transfected with the scramble or sh-RUBCN-S, and then co-cultured with CD8^+^T cells, and cell viability was measured by CCK-8. **(N)** The expression levels of MHC-I (HLA-A, -B, -C) in CUL5-KO T24 cells co-cultured with CD8^+^ T cells were detected by western blotting. Data are presented as the means ± SD from three independent experiments. Student *t* test was applied to analyze and compare the data in C, D, K, and M. ns, nonsignificant; **P < 0.01; ***P < 0.001. The raw data underlying all figures can be found in S1 Data. Original blots and gels can be found in S1 Raw Images.

Rubicon (encoded by RUBCN) is initially identified as a negative regulator of autophagy, inhibiting the fusion of autophagosomes and lysosomes. [26,27] In order to understand the functions of the two RUBCN isoforms in regulating autophagy, we overexpressed either Rubicon-L or Rubicon-S in bladder cancer cells and assessed their respective effects on autophagy. Specifically, overexpression of Rubicon-S in CUL5-WT cells resulted in increased levels of SQSTM1/p62 and LC3 II, whereas overexpression of Rubicon-L had no effect on the levels of p62 and LC3 II (Figs 5A and S3A). Additionally, autophagic flux assays showed that overexpressed Rubicon-S blocked the fusion of autophagosomes and lysosomes, thereby inhibiting autophagy and leading to an increase in immature autophagosomes (yellow spots), while Rubicon-L did not exhibit this function (Figs 5B and S3B). Subsequently, we examined whether both RUBCN isoforms affect the sensitivity of bladder cancer cells to CD8^+^ T cell-mediated killing. Notably, overexpression of Rubicon-S significantly increased the sensitivity of bladder cancer cells to CD8^+^ T cell-mediated killing, but overexpression of Rubicon-L had no effect on the viability of bladder cancer cells (Figs 5C and S3C). Furthermore, we knocked down RUBCN-L and RUBCN-S in bladder cancer cells, respectively, and found that RUBCN-S knockdown promoted autophagy in bladder cancer cells (Figs 5D and S3D). Moreover, we discovered that Rubicon-S interacted with the class III phosphatidylinositol-3 kinase complex II (PI3KC3-CII), which is required for autophagosome maturation and contains UVRAG and Beclin1, whereas Rubicon-L failed to bind to the PI3KC3-CII (Figs 5E, 5F, S3E, and S3F). These results indicated that Rubicon-S inhibited autophagy by interacting with the PI3KC3-CII.

### CUL5 knockout increases the sensitivity of bladder cancer cells to CD8^+^ T cell-mediated killing through inhibition of autophagy

We further explored how CUL5-KO increased the sensitivity of bladder cancer cells to CD8^+^ T cell-mediated killing. According to the WB and autophagic flux assays, we observed that autophagy was significantly inhibited in CUL5-KO cells (Figs 5G, 5H, S3G, and S3H). Next, we demonstrated that knockdown of PTBP1 in bladder cancer cells reversed the autophagy inhibition mediated by CUL5-KO (Figs 5I and S3I). Intriguingly, knockdown of RUBCN-L in CUL5-KO cells did not induce any significant changes in autophagy (Figs 5J and S3J) and had no effect on the viability of CUL5-KO cells (Figs 5K and S3K). In contrast, knockdown of RUBCN-S in bladder cancer cells led to the reversal of autophagy inhibition caused by CUL5-KO (Figs 5L and S3L), and restored resistance to CD8^+^ T cell-mediated killing (Figs 5M and S3M). Taken together, these findings suggested that CUL5 knockout increased the sensitivity of bladder cancer cells to CD8^+^ T cell-mediated killing via inhibition of autophagy. In previous studies, several different mechanisms have been proposed of how autophagy could modulate immune evasion [28–30]. To further address this question, we measured the expression of MHC-I (HLA-A, -B, -C). In CUL5-KO cells co-cultured with CD8^+^ T cells, MHC-I expression was significantly increased (Fig 5N). These findings suggested that CUL5 loss suppressed autophagy and elevated MHC-I, thereby enhancing the sensitivity of bladder cancer cells to CD8^+^ T cell–mediated killing. Additionally, we found that CUL5 loss in the lung cancer cell line A549 also leads to increased RUBCN-S levels, thereby inhibiting autophagy and increasing the sensitivity of lung cancer cells to CD8^+^ T cell-mediated killing (S4A–S4C Fig).

### CUL5 loss and inhibition of autophagy enhances the efficacy of PD-1 blockade therapy in bladder cancer

**Fig 6:**
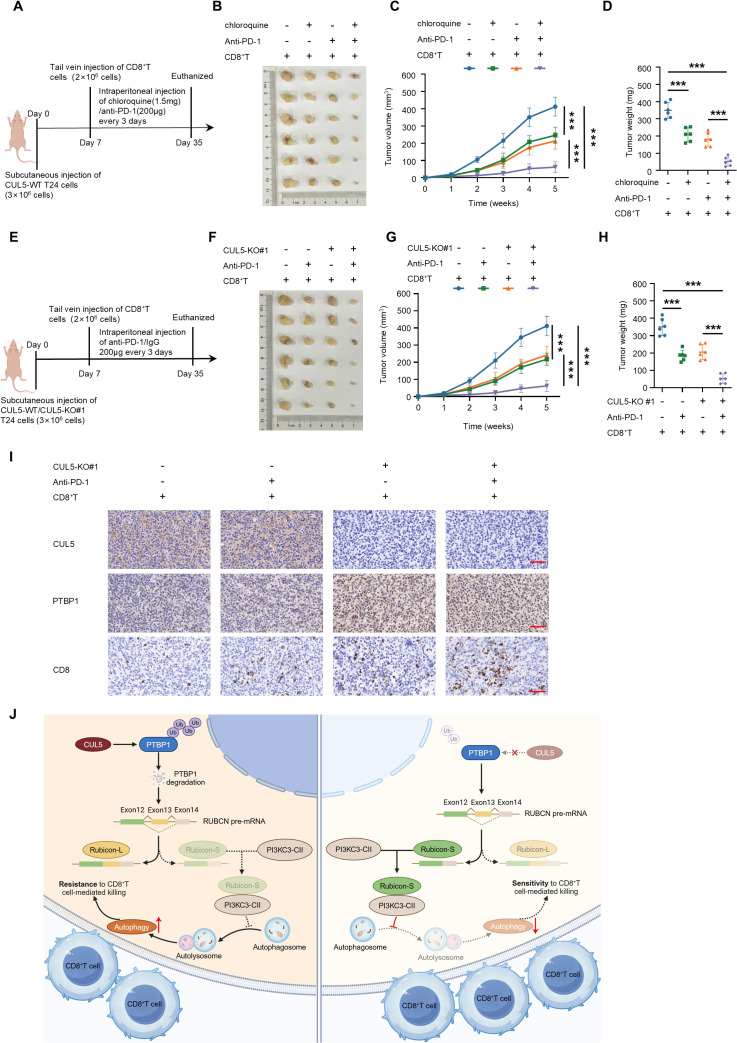
CUL5 loss and inhibition of autophagy enhance the efficacy of PD-1 blockade therapy in bladder cancer. **(A)** Schematic diagram of in vivo chloroquine and anti-PD-1 therapy. **(B)** The tumor-bearing ability of different experimental groups was evaluated by a xenograft mouse model (*n* = 6). **(C)** The tumor volumes at the indicated weeks after cell inoculation were measured (*n* = 6). **(D)** The mice were euthanized after the last measurement, and the tumors were harvested and weighed (*n* = 6). **(E)** Schematic diagram of in vivo anti-PD-1 therapy. **(F)** The tumor-bearing ability of different experimental groups was evaluated by a xenograft mouse model (*n* = 6). **(G)** The tumor volumes at the indicated weeks after cell inoculation were measured (*n* = 6). **(H)** The mice were euthanized after the last measurement, and the tumors were harvested and weighed (*n* = 6). **(I)** Immunohistochemical images of CUL5, PTBP1, and CD8 in the subcutaneous tumors. Bar: 50 μm. **(J)** Model describing the effect of CUL5 on the sensitivity of bladder cancer cells to CD8^+^ T cell-mediated killing. CUL5-KO could reduce the ubiquitination of PTBP1, thereby increasing the alternative splicing of RUBCN pre-mRNA, leading to an increase in Rubicon-S which inhibits autophagy, and thus enhances the sensitivity of bladder cancer cells to CD8^+^ T cell-mediated killing (The cartoon was created in BioRender. Naiwei, Z. (2025) https://BioRender.com/glislir). Data are presented as the means ± SD. ANOVA was applied to analyze and compare the data in C and G. Student *t* test was applied to analyze and compare the data in D and H. ***P < 0.001. The raw data underlying all figures can be found in S1 Data.

Given that CUL5-KO increased the sensitivity of bladder cancer cells to CD8^+^T cell-mediated killing by inhibiting autophagy, we subsequently investigated its role in regulating sensitivity to anti-PD-1 therapy. We selected the autophagy inhibitor chloroquine, which inhibits the fusion of autophagosomes and lysosomes, for use in a bladder cancer xenograft mouse model (Fig 6A). Either chloroquine or anti-PD-1 monotherapy inhibited tumor growth to some extent, while the combination therapy with chloroquine significantly enhanced the sensitivity of anti-PD-1 therapy in bladder cancer (Fig 6B–6D). We also explored the potential synergistic therapeutic effect of CUL5-KO and anti-PD-1 on bladder cancer (Fig 6E). The combination of CUL5 deletion synergistically enhanced the efficacy of anti-PD-1 immunotherapy (Fig 6F–6H). Immunohistochemical staining confirmed that the anti-PD-1 antibody significantly increased CD8^+^ T cells within the tumors, and this increase was more pronounced in CUL5-KO tumors (Fig 6I).

The above results demonstrated that CUL5-KO inhibited autophagy in bladder cancer cells, thereby increasing their sensitivity to CD8^+^ T cell-mediated killing (Fig 6J). Furthermore, CUL5-KO or inhibition of autophagy enhanced the immunotherapeutic effect of anti-PD-1, providing a promising therapeutic strategy for bladder cancer patients.

## Discussion

Bladder cancer is one of the globally prevalent urologic malignancies that carries a heavy social burden [3,31]. Despite the remarkable clinical benefits of immune checkpoint inhibitors, only a subset of patients achieves long-term durable responses, which severely limits their clinical efficacy [3]. Therefore, elucidating the molecular mechanisms underlying immunotherapy resistance is crucial for the development of novel therapeutic strategies aimed at enhancing its clinical benefit. Herein, we conducted a whole-genome CRISPR-Cas9 screen under CD8^+^ T cells pressure and identified CUL5 as a key regulator of bladder cancer cells sensitivity to CD8^+^ T cell-mediated killing. As an important member of the cullin family of E3 ligases, CUL5 has been reported to have multiple substrates that exert different roles [15]. The CUL5 ubiquitin ligase complex mediates the resistance of lung cancer cells to CDK9 and MCL1 inhibitors and also contributes to hematopoietic stem cell maintenance and thrombosis prevention [32–34]. In our specific context, we observed that CUL5 knockout alone did not significantly alter the proliferation of bladder cancer cells in vivo or in vitro. However, the role of CUL5 in immune cells has been increasingly reported. For instance, CUL5 regulates the differentiation of CD4^+^ T cells [35], CUL5 knockout enhances the activity of CD8^+^ T cells [16], and promotes the efficacy of CAR T cells [36]. Nevertheless, the role of CUL5 in tumor cells for immunotherapy has not yet been fully elucidated. In this study, we found that knockout of CUL5 significantly promoted the sensitivity of bladder cancer cells to CD8^+^ T cell-mediated killing, thereby inhibiting cellular proliferation and tumor growth. More importantly, CUL5 loss increased the immunotherapeutic efficacy of bladder cancer, providing a potential therapeutic strategy for bladder cancer. For patients, defining a safe therapeutic window is crucial, which necessitates a systematic evaluation of on-target effects. Comprehensive preclinical verification, particularly using conditional knockout mouse models, is imperative to validate whether targeted CUL5 inhibition can enhance immunotherapeutic efficacy while minimizing off-target toxicity. Future translational efforts need to focus on tumor-specific or cell-type-specific delivery strategies. Together, these findings reveal a novel role for CUL5 in tumor immunity.

Autophagy is an important mechanism for promoting tumor immune evasion and inhibiting the efficacy of tumor immunotherapy [28,37]. Tumor cells express low levels of MHC-I, which enables escape from CD8^+^ T cell mediated killing. In pancreatic cancer, inhibiting autophagy promotes CD8^+^ T cell-mediated killing by restoring the expression of MHC-I molecules on the surface of tumor cells [28]. Another study reported that in pancreatic cancer, impaired autophagy promotes the lysis of tumor cells by CD8^+^ T cells through enhanced accumulation of granzyme B [29]. In colon and breast cancers, autophagy protects tumors from T cell-mediated killing by inhibiting TNF-α induced apoptosis, and the study confirmed in mouse tumor models that tumors with impaired autophagy displayed enhanced responsiveness to immune checkpoint inhibitors [30]. Nonetheless, the role of autophagy in bladder cancer immunotherapy has not been fully clarified. Our findings suggested that CUL5 loss suppressed autophagy and elevated MHC-I, thereby enhancing the sensitivity of bladder cancer cells to CD8^+^ T cell-mediated killing. Previous studies have reported that Rubicon acts as a negative regulator of autophagy by blocking autophagosome maturation [26,27]. However, a RUBCN isoform lacking the RUN domain has been shown to promote autophagy in B cells [38]. In this study, we identified a novel RUBCN-S isoform lacking exon 13 in bladder cancer cells. Specifically, Rubicon-S could bind to PI3KC3-CII, thereby inhibiting autophagy and increasing the sensitivity of bladder cancer cells to CD8^+^ T cell-mediated killing, whereas Rubicon-L could not bind to the PI3KC3-CII and therefore lacked such function. These results enhance our understanding of the role of different RUBCN isoforms in regulating autophagy in tumor cells. However, the correlation between RUBCN-S levels in tumors and autophagy activity and response to immunotherapy requires further investigation in clinical samples. Despite that, we revealed that knockout of CUL5 increased the sensitivity of bladder cancer cells to CD8^+^ T cell-mediated killing by inhibiting autophagy. This was consistent with previous findings that inhibition of autophagy increased the antitumor effects of CD8^+^ T cells [29,30]. Notably, our in vivo study showed that chloroquine enhanced the efficacy of anti-PD-1 therapy, which provided further evidence that inhibition of autophagy improved the efficacy of tumor immunotherapy.

AS is a vital post-transcriptional process that drives the diversity of the transcriptome and proteome in eukaryotes [39,40]. According to our research, we discovered that the expression of the RUBCN isoforms was regulated by splicing factor PTBP1, and we demonstrated that the RRM3-4 tandem domain of PTBP1 played a critical role in RUBCN pre-mRNA AS. Importantly, PTBP1 was regulated by ubiquitination modifications. Previous findings have indicated that the E3 ubiquitin ligase TRIP12 promoted ubiquitination and degradation of PTBP1 in non-small cell lung cancer [41]. Recent studies propose that CUL5 regulates autophagy through p62 or by modulating mTOR via SIN1 [42,43]. However, our IP-MS analysis did not detect interactions between CUL5 and p62 or SIN1. This study showed that PTBP1 interacted with CUL5 and served as a previously unrecognized ubiquitination substrate of CUL5. Therefore, we speculate that the distinct characteristics of CUL5 may be attributed to its different substrates and cellular specificity. Notably, our study also demonstrated that PTBP1 interacted with the CUL5 through its RRM2 domain, leading to its ubiquitination. This finding further highlights the distinct functional roles of different RRM domains in PTBP1.

In summary, the new insights generated from this study enhance our understanding of immune evasion in bladder cancer. We elucidate a novel mechanism whereby the knockout of CUL5 in bladder cancer cells reduces the ubiquitination of PTBP1, thereby regulating the AS of RUBCN pre-mRNA to increase the RUBCN-S isoform, which in turn inhibits autophagy and increases sensitivity to CD8^+^ T cell-mediated killing. Our findings suggest that targeting the CUL5/PTBP1/RUBCN-S/autophagy pathway is a promising therapeutic strategy to enhance the efficacy of bladder cancer immunotherapy.

## Materials and methods

### Cell culture and reagents

Human bladder cancer cell lines T24 and UMUC3, lung cancer cell line A549, alone with human embryonic kidney (HEK) 293T cells, were purchased from American Type Culture Collection (ATCC, USA). T24 cells and A549 cells were cultured in RPMI-1640 medium (Gibco, USA) supplemented with 10% FBS (Gibco, Australia origin), 1% penicillin/streptomycin (Gibco, USA). UMUC3 cells and 293T cells were cultured in DMEM (Gibco, USA) supplemented with 10% FBS (Gibco, Australia origin), 1% penicillin/streptomycin (Gibco, USA). Cells were cultured in an incubator at 37 °C with humidified atmosphere of 5% CO_2_. All cell lines were verified within 6 months prior to use through short tandem repeat profiling and confirmed negative for Mycoplasma contamination.

### Genome-wide CRISPR-Cas9 knockout screen

T24 cells were transduced with the GeCKO v2 library with a low MOI of 0.3. Transduced cells were selected with puromycin (1 μg/mL) for 7 days. Then, the cells were either cultured in the absence of effectors or co-cultured with activated CD8^+^ T cells at an effector to target (E:T) ratio of 3:1. Following a 24 h co-culture period, cells were washed twice with PBS and allowed to recover for an additional 24 h. The surviving cells were harvested and genomic DNA was isolated. The sgRNA sequences were amplified using NEBNext High-Fidelity 2X PCR Master Mix (NEB, M0541) and subjected to next generation sequencing (NGS) by Novogene Technology (Beijing, China). Changes in sgRNA abundance were compared between bladder cancer cells challenged and unchallenged by CD8^+^ T cells. The MAGeCK v0.5.7 algorithm was used to process and analyze the CRISPR screen data [44].

### CRISPR-Cas9 knockout

The sgRNAs for the CUL5 gene (S2 Table) were designed using the web tool CHOPCHOP (http://chopchop.cbu.uib.no) and were synthesized by TSINGKE (Wuhan, China). The designed CUL5 sgRNAs were cloned into the lentiCRISPR v2 vector (Sigma-Aldrich). HEK293T cells were seeded in a 100 mm culture dish the day before transfection. After 24 h, the cells were co-transfected with lentiCRISPR v2 vector containing CUL5-specific sgRNA (9 μg), along with lentiviral packaging plasmids psPAX2 (6 μg) and the envelope plasmid pMD2.G (3 μg), using Lipofectamine 3000 (Invitrogen, USA) according to the manufacturer’s protocol. Six to eight hours later, the medium was replaced with fresh DMEM (Gibco, USA) medium. Lentiviral supernatant was collected 48 h post transfection, then filtered through a 0.45 μm filter, and purified. Bladder cancer cells were transfected following the virus resuspension. Transfected T24 or UMUC3 cells were selected in media containing 1 μg/mL puromycin (Invitrogen, USA) for 7 days, and then inoculated into 96-well plates to picked single-cell clones. Deletion efficiency was verified by western blotting with the anti-CUL5 antibody (A5369, Abclonal).

### Plasmids construction and cell transfection

To construct CUL5 and PTBP1 overexpression plasmids, human CUL5 and PTBP1 cDNAs were synthesized by TSINGKE (Wuhan, China) and cloned into pcDNA3.1-3*Flag-C vector (Sigma-Aldrich, USA). The short hairpin RNAs (shRNAs) targeting PTBP1, RUBCN-L, and RUBCN-S (S2 Table) were synthesized by TSINGKE Biotech (Wuhan, China), and then cloned into the pLKO.1 vector (Sigma-Aldrich, USA). Truncations of PTBP1 were amplified with indicated primers (S2 Table), and were cloned into the pcDNA3.1-3*Flag-C vector (Sigma-Aldrich, USA). HA-Ub plasmid as well as its lysine site mutation plasmid, and Rubicon overexpression plasmid were purchased from Paivibio (Wuhan, China). Neofect DNA transfection reagent (Neofect biotech, Beijing, China) was used for plasmid transient transfection according to the manufacturer’s instructions.

### RNA preparation and qRT-PCR

Total RNA was extracted from the cell lines using TRIzol reagent (Invitrogen, USA) following the manufacturer’s instructions. This RNA was then reverse transcribed to cDNA using HiScript III RT SuperMix for qPCR (Vazyme, China). Real-time PCR reactions were conducted with SYBR Green Master Mix (Vazyme, China). The results were analyzed using the StepOne Plus Real-Time PCR System (Applied Biosystems, USA), employing the 2^−ΔΔCt^ method to interpret the transcript level findings. For AS analysis of RUBCN isoforms, the conventional PCR was performed using the PrimeSTAR GXL Premix (Takara Bio, Japan) and the indicated primers. The RUBCN-L and RUBCN-S PCR products were loaded onto 2.5% agarose gels for electrophoresis and visualized by the BLT GelView 1500Plus Smart UV Gel Imaging System (Guangzhou, China). All primers sequences were listed in S2 Table.

### RNA sequencing

Total RNA was extracted from both CUL5 knockout T24 cells and their corresponding control cells using TRIzol reagent (Invitrogen, USA) according to the manufacturer’s instructions. After that, transcriptome sequencing was performed by SeqHealth (Wuhan, China). Gene expression levels were normalized using the Reads Per Kilobase Million method. AS events, including SE, alternative 3′ splice site (A3SS), alternative 5′ splice site (A5SS), mutually exclusive exon (MXE), and retained intron (RI), were quantified through replicate Multivarite Analysis of Transcript Splicing [45]. The detected differential AS genes were subjected to KEGG enrichment using a P value less than 0.05 as a screening criterion. The RNA sequencing data were deposited in the Gene Expression Omnibus (GEO) database: GSE279149.

### Western blotting

Total protein was extracted from cells using Radio immunoprecipitation assay (RIPA) lysis buffer (Servicebio, China) supplemented with protease inhibitor cocktail (MCE, China) and PMSF (Servicebio, China). The concentration of total protein was measured by BCA protein assay kit (HYCEZMBIO, China). Total protein was separated by SDS-PAGE and transferred to Polyvinylidene fluoride membranes (Millipore). After blocking with 5% non-fat milk for 1 h at room temperature, the membranes were washed three times with tris-buffered saline with tween-20 (TBST), each for 10 min, and then incubated with primary antibodies overnight at 4 °C. On the next day, the membranes were incubated with specific horseradish peroxidase (HRP)-conjugated secondary antibodies for one hour at room temperature. All membranes were visualized using an ECL substrate kit (HYCEZMBIO, China), and the resulting images were captured by the Alliance Q9 chemiluminescence imaging system (UVITEC, England). Antibodies used included primary antibodies against CUL5 (Abclonal, A5369), β-Actin (Proteintech, 66009-1-Ig), PRMT5 (Proteintech, 18436-1-AP), THOC2 (Proteintech, 55178-1-AP), THRAP3 (Proteintech, 19744-1-AP), SNRNP200 (Proteintech, 23875-1-AP), PTBP1 (Proteintech, 12582-1-AP), SF3B1(Proteintech, 27684-1-AP), PRPF8(Proteintech, 11171-1-AP), SF3B2 (Proteintech, 10919-1-AP), HNRNPC (Proteintech, 11760-1-AP), LC3B (Abclonal, A5618), P62 (Proteintech, 18420-1-AP), Rubicon (Proteintech, 21444-1-AP), UVRAG (Proteintech, 29190-1-AP), Beclin1 (Proteintech, 11306-1-AP), HLA-class I (Proteintech, 15240-1-AP), Rabbit control IgG (Abclonal, AC005), Mouse control IgG (Abclonal, AC011), Mouse anti-HA tag (Abclonal, AE008), Rabbit anti-HA tag (Abclonal, AE036), Mouse anti-Flag tag (Abclonal, AE005), and Rabbit anti-Flag tag (Abclonal, AE004); HRP-conjugated secondary goat anti-mouse (Proteintech, SA00001-1), or goat anti-rabbit (Proteintech, SA00001-2) antibodies. Original western blot images for all relevant figures are shown in S1 Raw Images.

### Isolation and culture of CD8^+^ T cells from peripheral blood

The blood was obtained from volunteers, and human peripheral blood mononuclear cells were isolated and purified by using the Ficoll kit (TBD, CHINA) following the manufacture’s instruction. Then, CD8^+^ T cells were purified using human CD8 MicroBeads (Miltenyi, Germany). Human CD8^+^ T cells were cultured at a concentration of 2 × 10^6^ cells/mL in RPMI-1640 medium (Gibco, USA) supplemented with 10% human AB serum (Gibco, USA), 1% penicillin/streptomycin (Gibco, USA), and recombinant human IL-2 (100 U/mL) (Biolegend, USA). Isolated CD8^+^ T cells were stimulated for 48 h in 2 µg/mL plate-bound anti-human CD3 (Biolegend, USA) and 2 µg/mL soluble anti-human CD28 (Biolegend, USA).

### Cell counting kit-8 assay

T24 and UMUC3 cells were seeded in 96-well plates at a density of 3 × 10³ cells per well and subjected to cultivation over various durations (0, 24, 48, and 72 h). The viability of these cells was then assessed using the CCK-8 assay kit (HYcezmbio, China), adhering to the manufacturer’s recommended procedures. For CD8^+^ T cells cytotoxicity assay, bladder cancer cells were seeded in 96-well plates for overnight incubation, and then co-cultured with CD8^+^T cells at an E: T ratio of 3:1. After 24 h of co-culturing, the bladder cancer cells were washed twice with PBS to remove CD8^+^ T cells and dead bladder cancer cells. Then, 110 μL of medium mixture containing CCK-8 solution (10 μL) was added into each well and incubated at 37 °C for 2 h. Cell viability was determined based on the optical density at 450 nm measured using an automatic microplate reader (TECAN, Infinite F50, Switzerland).

### Co-Immunoprecipitation (Co-IP)

Cells were lysed in 2 mL NP-40 Lysis Buffer (Beyotime, China) supplemented with protease inhibitor cocktail (MCE, China) and PMSF (Servicebio, China) for an hour on ice. Five percent of cell lysate was used as the Input group, and the remaining cell lysate was equally divided into two portions and incubated with 4 μg of primary antibodies or IgG antibody overnight at 4 °C, respectively. Then, Protein A/G Magnetic Beads (MCE, China) were incubated with cell lysate for 3 h at 4°C. The immunoprecipitated complexes were obtained after washing the magnetic beads five times. Afterward, these complexes underwent either western blot analysis or mass spectrometry for further investigation. The antibodies used are described in western blotting.

### Silver staining and mass spectrometry analysis

After electrophoresis was completed, the 7.5% SDS-PAGE gels were stained using the PAGE Gel Silver Staining Kit (Beyotime, China). Subsequently, mass spectrometry analysis was performed by APTBIO (Shanghai, China). The differential proteins identification and quantification were carried out using Proteome Discoverer software (version 1.4, Thermo Fisher Scientific).

### Ubiquitination assays

For ubiquitination assays, the HA-Ubiquitin plasmid or its lysine site mutant plasmid was transfected into either CUL5-WT or CUL5-KO bladder cancer cells. After 24 h, cells were incubated with 10 μM MG132 (MCE, China) for 24 h. Then, the Co-IP assay was performed as described above. After elution of antigen/antibody complexes, the ubiquitination level of PTBP1 was detected by WB. β-Actin protein levels were also determined in whole-cell lysates.

### RNA immunoprecipitation (RIP) assay

RIP assays were carried out utilizing the Magna RIP RNA-Binding Protein Immunoprecipitation Kit (Millipore, USA), adhering strictly to the manufacturer’s protocol. In brief, T24 and UMUC3 cells were lysed using RIP lysis buffer, which included an RNase inhibitor and a protease inhibitor cocktail (MCE, China). Then, the PTBP1 antibody or a rabbit IgG antibody was mixed with Protein A/G Magnetic Beads (MCE, China) and incubated with cell lysates at 4 °C overnight. The RNAs were extracted and purified utilizing proteinase K, and then quantified by qRT-PCR. Normal rabbit IgG was used as the negative control.

### Autophagic flux measurement

Autophagic flux was detected by tandem mCherry-EGFP-LC3B (Paivibio, Wuhan, China) immunofluorescence. Cells were transfected with mCherry-EGFP-LC3B. After transfection for 48 h, cells were harvested and re-seeded on confocal culture dish. The images were acquired with a Nikon A1Si Laser Scanning Confocal Microscope (Nikon Instruments, Japan). The colocalization of EGFP and mCherry puncta was examined. The autophagosomes with yellow puncta and autolysosomes with red puncta.

### Tumor xenograft assay

All procedures for the animal experiments were approved by the Institutional Animal Care and Use Committee (IACUC No.3133) of Huazhong University of Science and Technology (Wuhan, China). Female BALB/c nude mice, aged four weeks, were selected for tumor xenograft studies. The animals were randomly allocated to either the experimental or control group, with six mice per group. Blinding was not employed in these experiments. CUL5-wild type (WT) or knockout (KO) bladder cancer cells (3 × 10^6^) were subcutaneously injected into the right flank of the nude mice. One week after the bladder cancer cells were injected, the experimental group was injected with activated CD8^+^T cells (2 × 10^6^) isolated from volunteers’ peripheral blood via the tail vein. Tumor growth rates were monitored every other week. Tumor volumes were determined using the formula: Volume = (*π*/6) × Length × (Width^2^). Upon completion of the experiment, the mice were euthanized by cervical dislocation, and the tumors were excised and weighed.

For Chloroquine and anti-PD-1 therapy, nude mice injected with CUL5-WT T24 cells (3 × 10^6^ cells on each side of mouse). After 1 week, activated CD8^+^ T cells (2 × 10^6^ cells) isolated from volunteers’ peripheral blood were injected via the tail vein. Tumor-bearing mice were injected intraperitoneally every three days with anti-PD-1 antibody (200 µg per mouse) and/or chloroquine (1.5 mg per mouse), and IgG antibody or DMSO was used as control at similar dosage and frequency.

For anti-PD-1 therapy, nude mice injected with CUL5-WT or CUL5-KO#1 T24 cells (3 × 10^6^ cells on each side of mouse). After 1 week, activated CD8^+^ T cells (2 × 10^6^ cells) isolated from volunteers’ peripheral blood were injected via the tail vein. Tumor-bearing mice received intraperitoneal injections of an anti-PD-1 antibody at a dose of 200 μg per mouse every three days. As a control, mice were injected with an IgG antibody at the same dosage and frequency. All animal procedures adhered to the NIH guidelines for the Care and Use of Laboratory Animals.

### Immunohistochemical staining

The paraffin-embedded sections of tumor tissues underwent dewaxing, antigen repair, and then incubated with specific primary antibodies (anti-CUL5, anti-PTBP1, and anti-CD8) at 4 °C overnight. Then, the sections were incubated with a secondary antibody for 1 h at room temperature and stained with DAB reagent. IHC staining evaluation was performed using CaseViewer software.

### Statistical analysis

Continuous variables were indicated as mean ± standard deviation (SD). Statistical analysis was performed using GraphPad Prism 9.5 software (GraphPad software, San Diego, CA). Student *t* test and ANOVA were used to assess the group difference. *P* < 0.05 was considered statistically significant.

### Ethics statement

Ethics approval for animal work was provided by the Institutional Animal Care and Use Committee (IACUC) of Huazhong University of Science and Technology. IACUC Number is 3133.

## Supporting information

S1 Fig CUL5 interacts with PTBP1 protein. (**A**) Biological processes enrichment in the protein-protein interaction (PPI) network predicted by the STRING database (https://cn.string-db.org/). (**B**) Nine proteins that were primarily enriched in the RNA splicing biological process within the PPI network.
(TIF)

S2 Fig Loss of CUL5 regulates RUBCN alternative splicing by PTBP1. (**A**) Agarose gel electrophoresis analysis of the RUBCN isoforms in CUL5-KO UMUC3 cells. The structure of each PCR product was indicated schematically on the right, and the alternative exon affected by CUL5 was painted in orange. (**B**) Agarose gel electrophoresis analysis of the RUBCN isoforms in PTBP1-overexpressed UMUC3 cells. The efficiency of PTBP1-overexpressed in UMUC3 cells was detected by western blotting (left) and qRT-PCR (right). (**C**) Agarose gel electrophoresis analysis of the RUBCN isoforms in PTBP1-knockdown UMUC3 cells. The efficiency of PTBP1-knockdown in UMUC3 cells was detected by western blotting (left) and qRT-PCR (right). (**D**) Western blotting with the indicated antibodies in CUL5-WT and CUL5-KO UMUC3 cells transfected with scramble or sh-PTBP1#1, and agarose gel electrophoresis for analysis of RUBCN isoforms. (**E**) Agarose gel electrophoresis analysis of the RUBCN isoforms in CUL5-KO UMUC3 cells co-cultured with CD8^+^ T cells. (**F**) Agarose gel electrophoresis analysis of the RUBCN isoforms in PTBP1-overexpressed UMUC3 cells co-cultured with CD8^+^ T cells. (**G**) Agarose gel electrophoresis analysis of the RUBCN isoforms in PTBP1-knockdown UMUC3 cells co-cultured with CD8^+^ T cells. (**H**) RIP assays in UMUC3 cells using PTBP1 and IgG antibody. The precipitate was subjected to Western blotting with the antibody against PTBP1. The PTBP1-enriched RUBCN pre-mRNA relative to the IgG-enriched value was calculated by qRT-PCR. (**I**) Agarose gel electrophoresis analysis of the RUBCN isoforms in UMUC3 cells transfected with vector, full-length or truncations of Flag-tagged recombinant PTBP1. Data are presented as the means ± SD from three independent experiments. Student *t* test was applied to analyze and compare the data in B, C, and H. ***P < 0.001. The raw data underlying all figures can be found in S1 Data. Original blots and gels can be found in S1 Raw Images.
(TIF)

S3 Fig CUL5 knockout increases the sensitivity of bladder cancer cells to CD8^+^ T cell-mediated killing by inhibiting autophagy. (**A**) The expression levels of autophagy marker LC3B and substrate P62 in UMUC3 cells transfected with vector, Flag-Rubicon-L or Flag-Rubicon-S were detected by western blotting. (**B**) UMUC3 cells were transfected with vector, Flag-Rubicon-L or Flag-Rubicon-S plasmids, and those co-transfected with mCherry-EGFP-LC3B. After 48 h expression, cells were harvested and re-seeded on confocal culture dish. The colocalization of EGFP and mCherry puncta was examined. The autophagosomes with yellow puncta and autolysosomes with red puncta. Bar: 10 μm. (**C**) UMUC3 cells were transfected with the vector, Flag-Rubicon-L, and Flag-Rubicon-S plasmids for 48 h, and then co-cultured with CD8^+^ T cells for 24 h, and cell viability was measured by CCK-8. (**D**) Western blotting with the indicated antibodies in UMUC3 cells transfected with scramble, sh-RUBCN-L or sh-RUBCN-S. The efficiency of RUBCN-L knockdown and RUBCN-S knockdown in UMUC3 cells was detected by agarose gel electrophoresis (left) and qRT-PCR (right). (**E**) Co-IP assay using antibody specific for Rubicon showed that Rubicon interacted with UVRAG and Beclin1 in UMUC3 cells. The precipitate was subjected to western blotting with the antibodies against Rubicon, UVRAG, and Beclin1. (**F**) Co-IP assay using antibody specific for Flag showed that Flag-Rubicon-S interacted with UVRAG and Beclin1(Right), while Flag-Rubicon-L could not bind to UVRAG and Beclin1 in UMUC3 cells (Left). The precipitate was subjected to western blotting with the antibodies against Flag, UVRAG, and Beclin1. (**G**) The expression levels of LC3B and P62 in CUL5-KO UMUC3 cells were detected by western blotting. (**H**) CUL5-WT, CUL5-KO#1, and CUL5-KO#2 UMUC3 cells were transfected with the mCherry-EGFP-LC3B. After 48 h expression, cells were harvested and re-seeded on confocal culture dish. The colocalization of EGFP and mCherry puncta was examined. The autophagosomes with yellow puncta and autolysosomes with red puncta. Bar: 10 μm. (**I**) Western blotting with the indicated antibodies in CUL5-WT and CUL5-KO UMUC3 cells transfected with scramble or sh-PTBP1#1. (**J**) Western blotting with the indicated antibodies in CUL5-WT and CUL5-KO UMUC3 cells transfected with scramble or sh-RUBCN-L, and agarose gel electrophoresis for analysis of RUBCN isoforms. (**K**) CUL5-WT, CUL5-KO#1, and CUL5-KO#2 UMUC3 cells were transfected with the scramble or sh-RUBCN-L for 48 h, and then co-cultured with CD8^+^ T cells for 24 h, and cell viability was measured by CCK-8. (**L**) Western blotting with the indicated antibodies in CUL5-WT and CUL5-KO UMUC3 cells transfected with scramble or sh-RUBCN-S, and agarose gel electrophoresis for analysis of RUBCN isoforms. (**M**) CUL5-WT, CUL5-KO#1, and CUL5-KO#2 UMUC3 cells were transfected with the scramble or sh-RUBCN-S for 48 h, and then co-cultured with CD8^+^ T cells for 24 h, and cell viability was measured by CCK-8. Data are presented as the means ± SD from three independent experiments. Student *t* test was applied to analyze and compare the data in C, D, K, and M. ns, nonsignificant; **P < 0.01; ***P < 0.001. The raw data underlying all figures can be found in S1 Data. Original blots and gels can be found in S1 Raw Images
(TIF)

S4 Fig CUL5 knockout increases the sensitivity of lung cancer cells to CD8^+^ T cell-mediated killing by inhibiting autophagy. (**A**) Western blotting with the indicated antibodies in CUL5-WT and CUL5-KO A549 cells transfected with scramble or sh-PTBP1#1, and agarose gel electrophoresis for analysis of RUBCN isoforms. (**B**) The expression levels of LC3B and P62 in CUL5-KO A549 cells were detected by western blotting. (**C**) CUL5-WT, CUL5-KO#1 and CUL5-KO#2 A549 cells were co-cultured with CD8^+^ T cells for 24 h, and cell viability was measured by CCK-8. Data are presented as the means ± SD from three independent experiments. Student *t* test was applied to analyze and compare the data in C. **P < 0.01. The raw data underlying all figures can be found in S1 Data. Original blots and gels can be found in S1 Raw Images
(TIF)

S1 Table Genes of CRISPR-Cas9 negative screening. We compared changes in sgRNA abundance between T24 cells challenged and unchallenged by CD8^+^ T cells, and then performed negative screening (*p* < 0.01, log_2_Fold change < −1.0, and good sgRNAs ≥ 5).
(XLSX)

S2 Table Primers, sgRNAs, and shRNA sequence. All sequence information was listed including primers for clone and quantitative RT-PCR, we also presented the sequences of sgRNAs and shRNAs used in this manuscript.
(XLSX)

S3 Table The proteins pulled down by CUL5 in T24-MS. Mass spectrometry to identify potential proteins that interact with CUL5. (XLSX)

S4 Table Types of alternative splicing in differential genes. We conducted high-throughput sequencing of RNA on the CUL5-WT and CUL5-KO T24 cells to analyze the types of alternative splicing.
(XLSX)

S1 Data All relevant data values for figures. (XLSX)

S1 Raw Images Unedited original Western blots and gels used in the study. (PDF)

## Abbreviations

A3SS: alternative 3′ splice site
A5SS: alternative 5′ splice site
AS: alternative splicing
Co-IP: Co-Immunoprecipitation
CUL5: Cullin5
HEK: human embryonic kidney
HRP: horseradish peroxidase
ICB: immune checkpoint blockade
MXE: mutually exclusive exon
PI3KC3-CII: Class III phosphatidylinositol-3 kinase complex II
PTBP1: polypyrimidine tract binding protein 1
RI: retained intron
RIPA: Radio immunoprecipitation assay
RNA-Seq: RNA sequencing
RRM: RNA recognition motif
RUBCN/Rubicon: RUN domain and cysteine-rich domain containing Beclin 1-interacting protein
SE: skipped exon
