# Supplementary figures and images for: CUL5 E3 ubiquitin ligase regulates the evasion of bladder cancer cells to CD8+ T cell-mediated killing by inhibiting autophagy
Source: PLoS Biol. 2026 Feb 9;24(2):e3003647. doi: 10.1371/journal.pbio.3003647 (PMC12900434; doi:10.1371/journal.pbio.3003647)

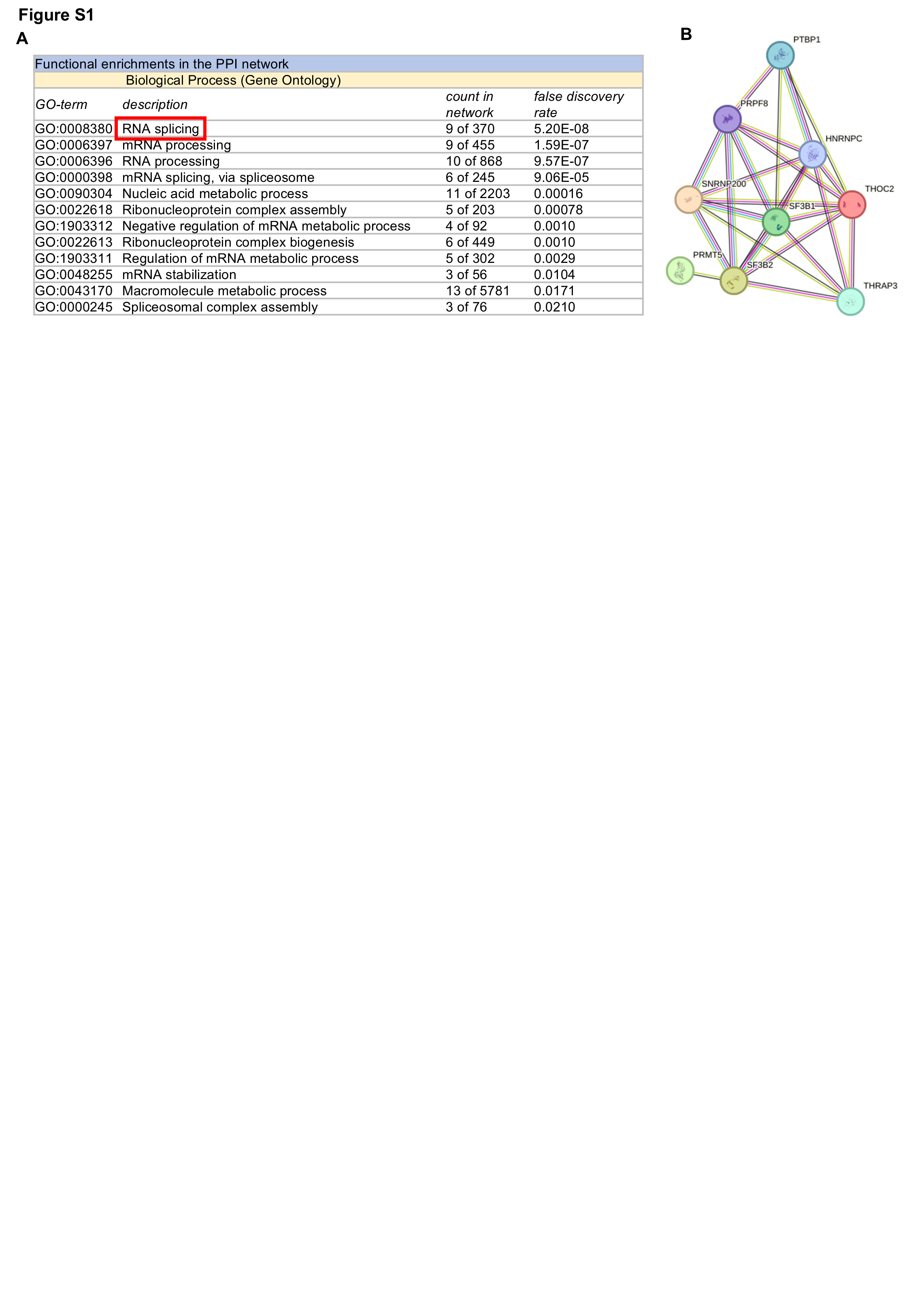

Supplement: S1 Fig — (A) Biological processes enrichment in the protein-protein interaction (PPI) network predicted by the STRING database (https://cn.string-db.org/). (B) Nine proteins that were primarily enriched in the RNA splicing biological process within the PPI network. (TIF) [file pbio.3003647.s001.TIF]

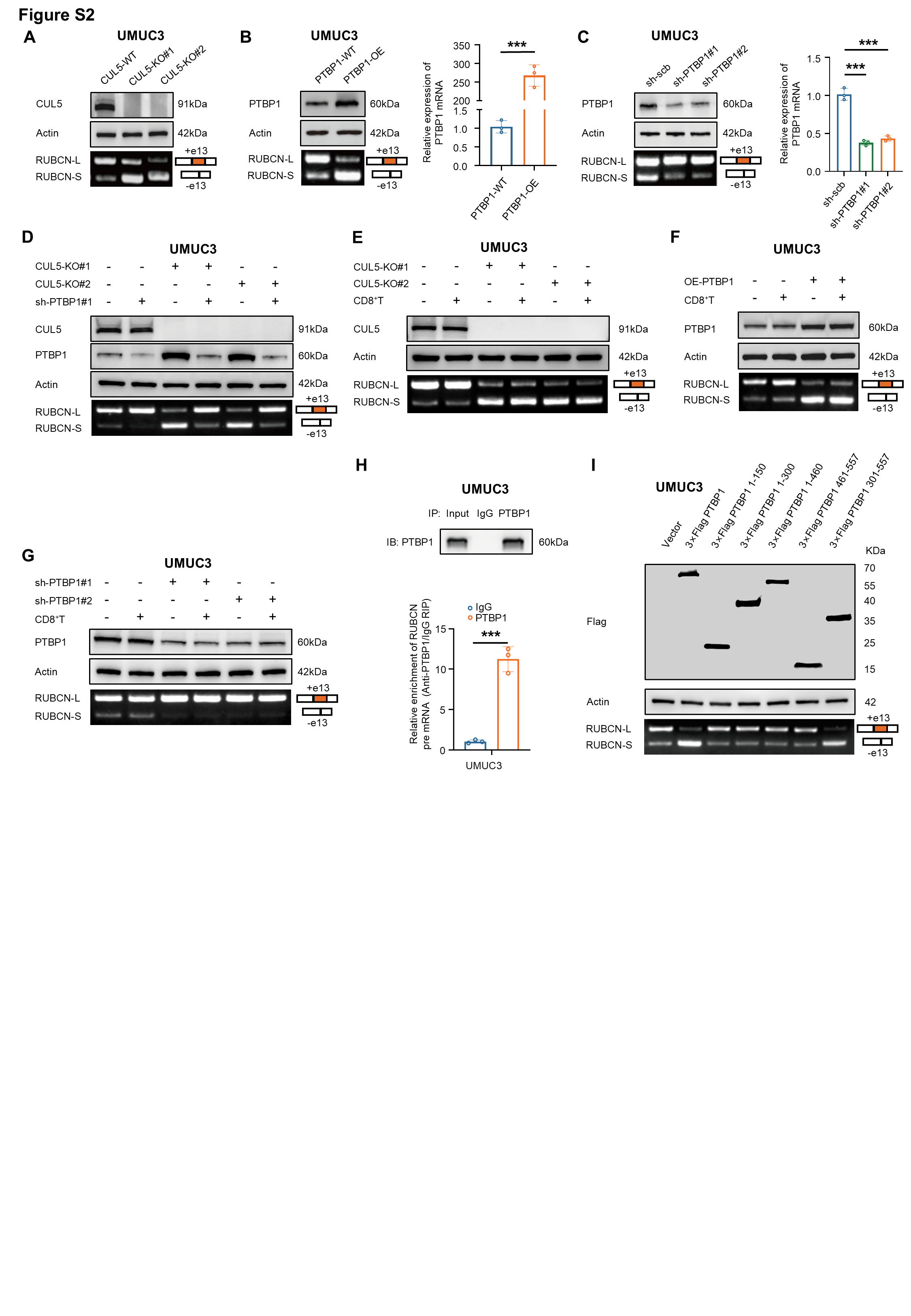

Supplement: S2 Fig — (A) Agarose gel electrophoresis analysis of the RUBCN isoforms in CUL5-KO UMUC3 cells. The structure of each PCR product was indicated schematically on the right, and the alternative exon affected by CUL5 was painted in orange. (B) Agarose gel electrophoresis analysis of the RUBCN isoforms in PTBP1-overexpressed UMUC3 cells. The efficiency of PTBP1-overexpressed in UMUC3 cells was detected by western blotting (left) and qRT-PCR (right). (C) Agarose gel electrophoresis analysis of the RUBCN isoforms in PTBP1-knockdown UMUC3 cells. The efficiency of PTBP1-knockdown in UMUC3 cells was detected by western blotting (left) and qRT-PCR (right). (D) Western blotting with the indicated antibodies in CUL5-WT and CUL5-KO UMUC3 cells transfected with scramble or sh-PTBP1#1, and agarose gel electrophoresis for analysis of RUBCN isoforms. (E) Agarose gel electrophoresis analysis of the RUBCN isoforms in CUL5-KO UMUC3 cells co-cultured with CD8+ T cells. (F) Agarose gel electrophoresis analysis of the RUBCN isoforms in PTBP1-overexpressed UMUC3 cells co-cultured with CD8+ T cells. (G) Agarose gel electrophoresis analysis of the RUBCN isoforms in PTBP1-knockdown UMUC3 cells co-cultured with CD8+ T cells. (H) RIP assays in UMUC3 cells using PTBP1 and IgG antibody. The precipitate was subjected to Western blotting with the antibody against PTBP1. The PTBP1-enriched RUBCN pre-mRNA relative to the IgG-enriched value was calculated by qRT-PCR. (I) Agarose gel electrophoresis analysis of the RUBCN isoforms in UMUC3 cells transfected with vector, full-length or truncations of Flag-tagged recombinant PTBP1. Data are presented as the means ± SD from three independent experiments. Student t test was applied to analyze and compare the data in B, C, and H. ***P < 0.001. The raw data underlying all figures can be found in S1 Data. Original blots and gels can be found in S1 Raw Images. (TIF) [file pbio.3003647.s002.TIF]

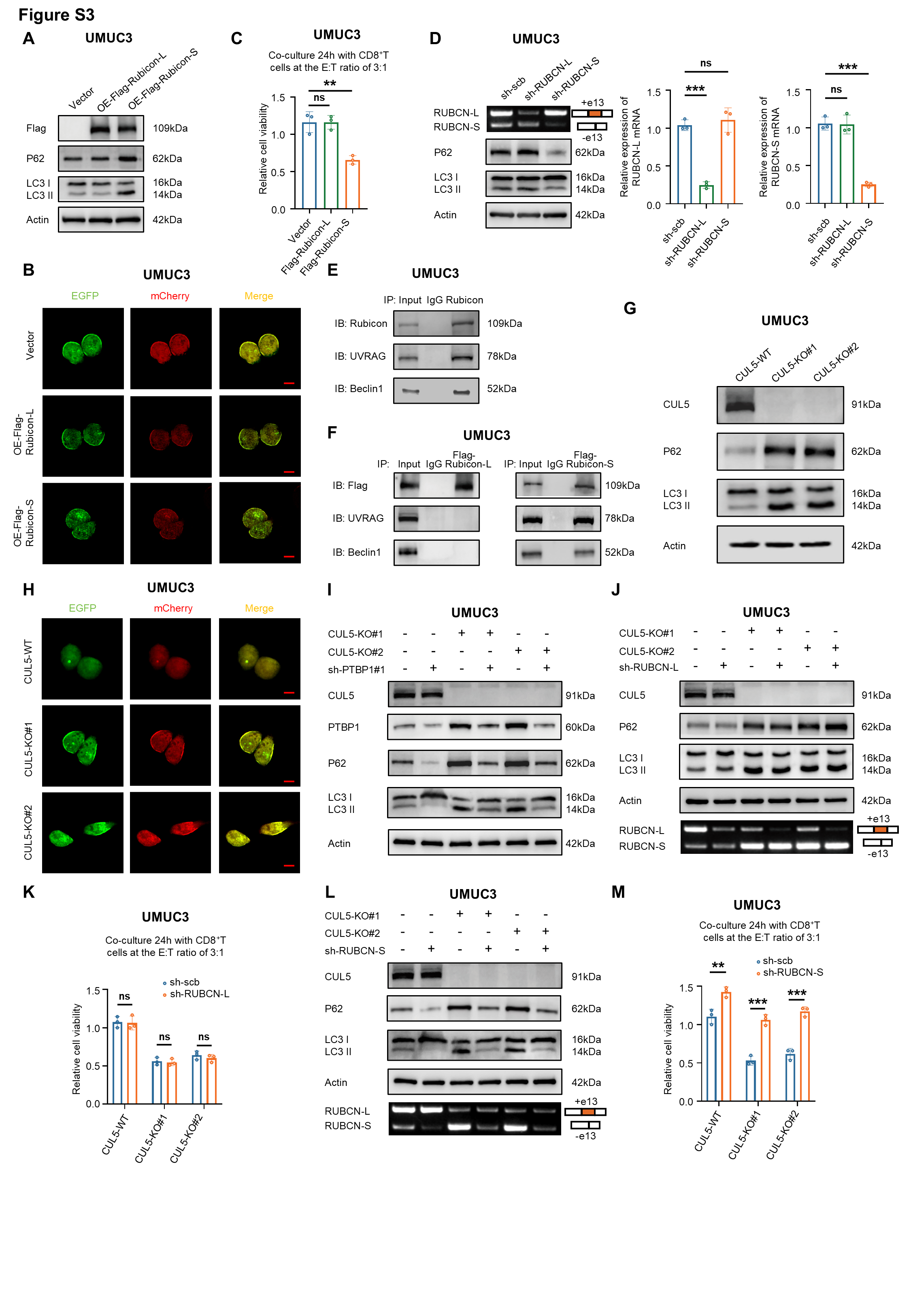

Supplement: S3 Fig — (A) The expression levels of autophagy marker LC3B and substrate P62 in UMUC3 cells transfected with vector, Flag-Rubicon-L or Flag-Rubicon-S were detected by western blotting. (B) UMUC3 cells were transfected with vector, Flag-Rubicon-L or Flag-Rubicon-S plasmids, and those co-transfected with mCherry-EGFP-LC3B. After 48 h expression, cells were harvested and re-seeded on confocal culture dish. The colocalization of EGFP and mCherry puncta was examined. The autophagosomes with yellow puncta and autolysosomes with red puncta. Bar: 10 μm. (C) UMUC3 cells were transfected with the vector, Flag-Rubicon-L, and Flag-Rubicon-S plasmids for 48 h, and then co-cultured with CD8+ T cells for 24 h, and cell viability was measured by CCK-8. (D) Western blotting with the indicated antibodies in UMUC3 cells transfected with scramble, sh-RUBCN-L or sh-RUBCN-S. The efficiency of RUBCN-L knockdown and RUBCN-S knockdown in UMUC3 cells was detected by agarose gel electrophoresis (left) and qRT-PCR (right). (E) Co-IP assay using antibody specific for Rubicon showed that Rubicon interacted with UVRAG and Beclin1 in UMUC3 cells. The precipitate was subjected to western blotting with the antibodies against Rubicon, UVRAG, and Beclin1. (F) Co-IP assay using antibody specific for Flag showed that Flag-Rubicon-S interacted with UVRAG and Beclin1(Right), while Flag-Rubicon-L could not bind to UVRAG and Beclin1 in UMUC3 cells (Left). The precipitate was subjected to western blotting with the antibodies against Flag, UVRAG, and Beclin1. (G) The expression levels of LC3B and P62 in CUL5-KO UMUC3 cells were detected by western blotting. (H) CUL5-WT, CUL5-KO#1, and CUL5-KO#2 UMUC3 cells were transfected with the mCherry-EGFP-LC3B. After 48 h expression, cells were harvested and re-seeded on confocal culture dish. The colocalization of EGFP and mCherry puncta was examined. The autophagosomes with yellow puncta and autolysosomes with red puncta. Bar: 10 μm. (I) Western blotting with the indicated an [file pbio.3003647.s003.TIF]

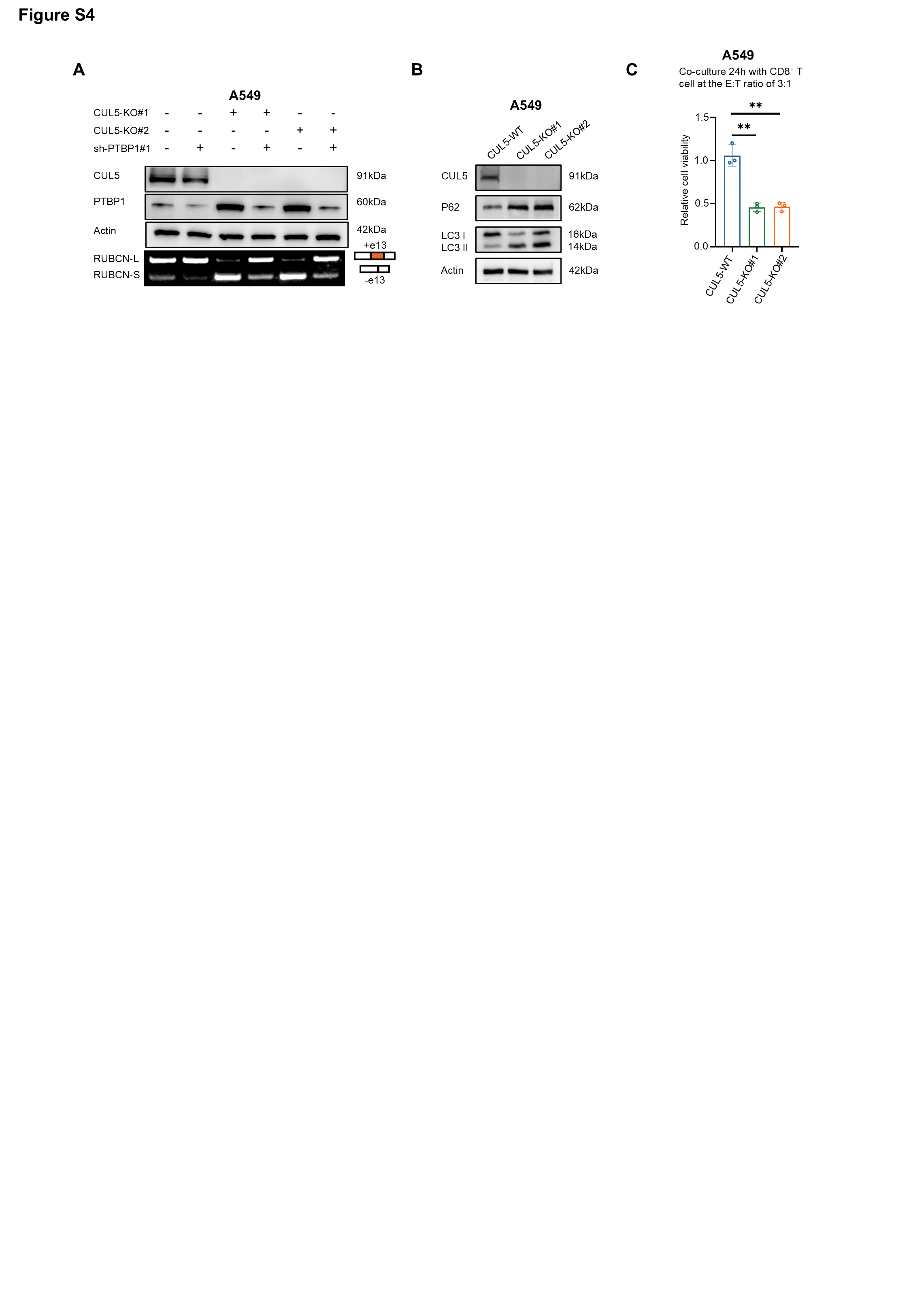

Supplement: S4 Fig — (A) Western blotting with the indicated antibodies in CUL5-WT and CUL5-KO A549 cells transfected with scramble or sh-PTBP1#1, and agarose gel electrophoresis for analysis of RUBCN isoforms. (B) The expression levels of LC3B and P62 in CUL5-KO A549 cells were detected by western blotting. (C) CUL5-WT, CUL5-KO#1 and CUL5-KO#2 A549 cells were co-cultured with CD8+ T cells for 24 h, and cell viability was measured by CCK-8. Data are presented as the means ± SD from three independent experiments. Student t test was applied to analyze and compare the data in C. **P < 0.01. The raw data underlying all figures can be found in S1 Data. Original blots and gels can be found in S1 Raw Images (TIF) [file pbio.3003647.s004.TIF]
