# Supplementary material for: CUL5 E3 ubiquitin ligase regulates the evasion of bladder cancer cells to CD8+ T cell-mediated killing by inhibiting autophagy
Source: PLoS Biol. 2026 Feb 9;24(2):e3003647. doi: 10.1371/journal.pbio.3003647 (PMC12900434; doi:10.1371/journal.pbio.3003647)

Original images of western blots

Figure 1C

T24

CUL5

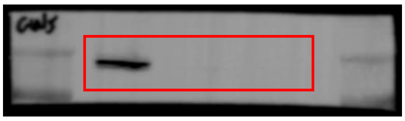

Actin

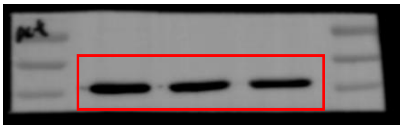

UMUC3

CUL5

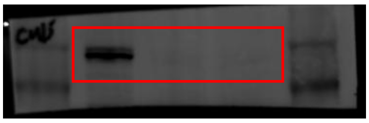

Actin

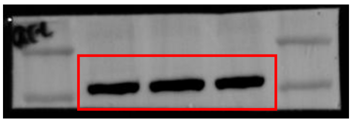

Figure 1F

T24

CUL5

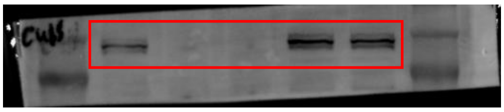

Actin

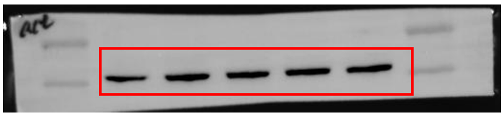

UMUC3

CUL5

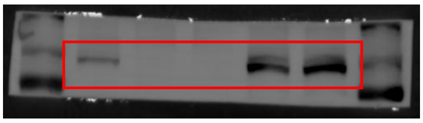

Actin

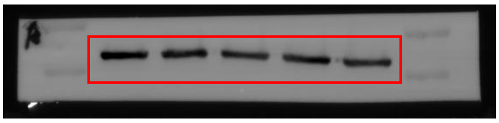

Figure 2A

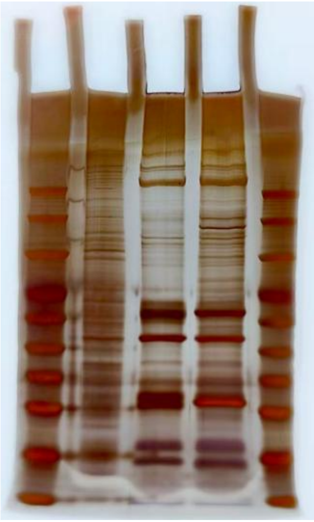

Figure 2C

T24

CUL5

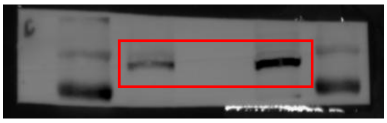

PRMT5

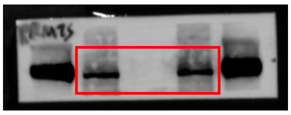

THOC2

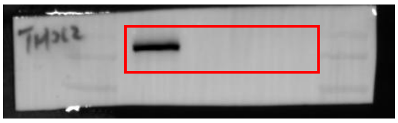

THRAP3

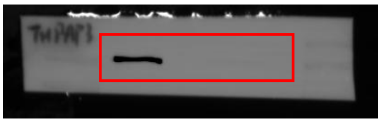

SNRNP200

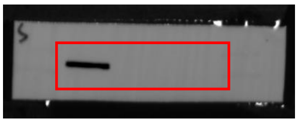

PTBP1

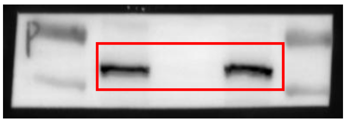

SF3B1

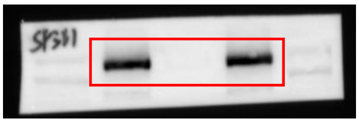

PRPF8

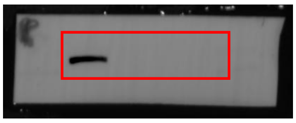

SF3B2

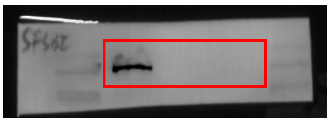

HNRNPC

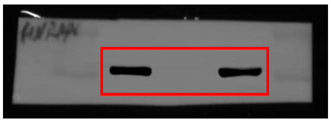

UMUC3

CUL5

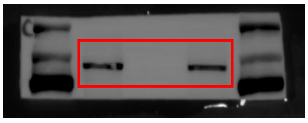

PRMT5

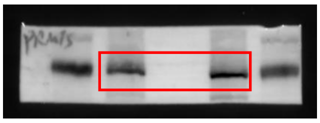

THOC2

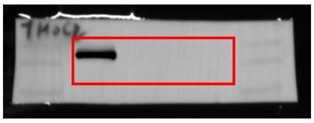

THRAP3

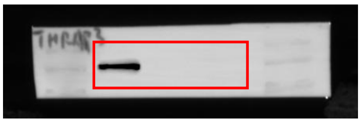

SNRNP200

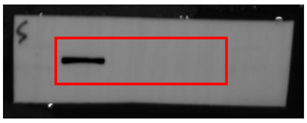

PTBP1

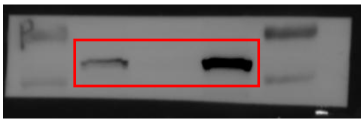

SF3B1

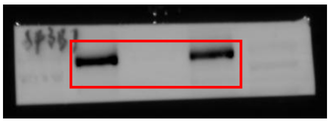

PRPF8

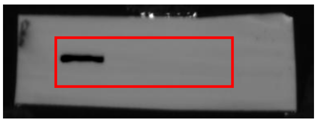

SF3B2

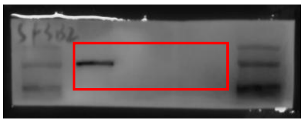

HNRNPC

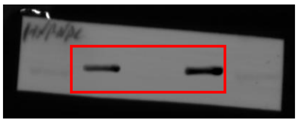

Figure 2D

CUL5

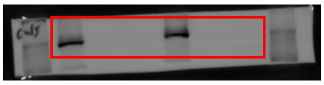

PRMT5

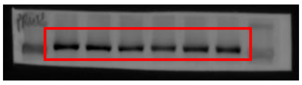

PTBP1

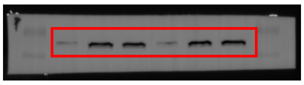

SF3B1

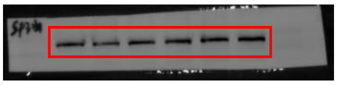

HNRNPC

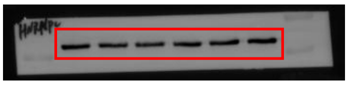

Actin

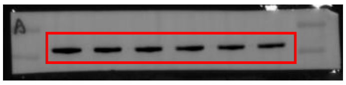

Figure 2E

T24

PTBP1

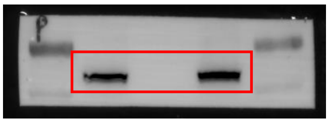

CUL5

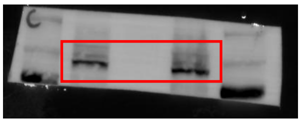

UMUC3

PTBP1

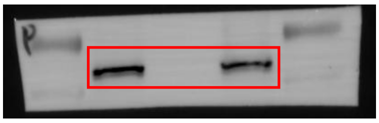

CUL5

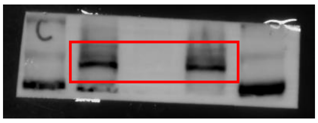

Figure 2F

T24

CUL5

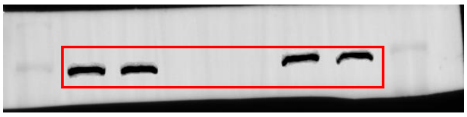

PTBP1

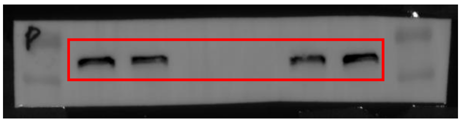

UMUC3

CUL5

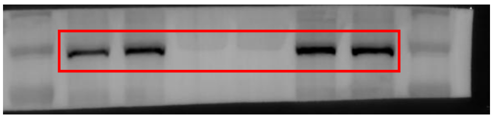

PTBP1

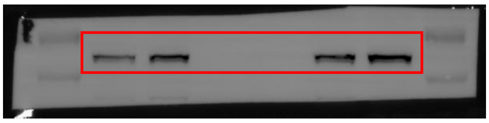

Figure 2H

Flag

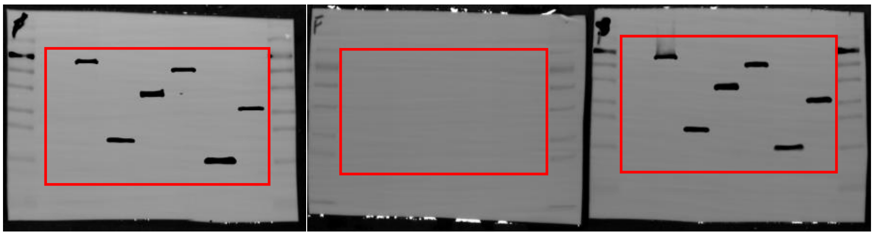

CUL5

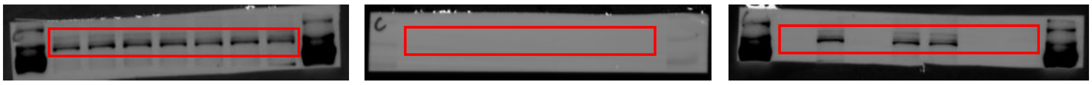

Actin

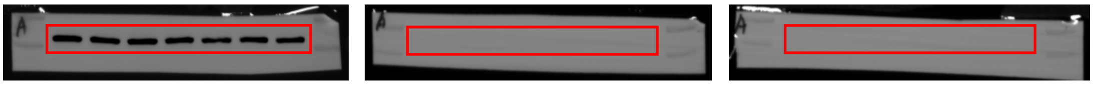

Figure 3A

T24

HA PTBP1 Actin

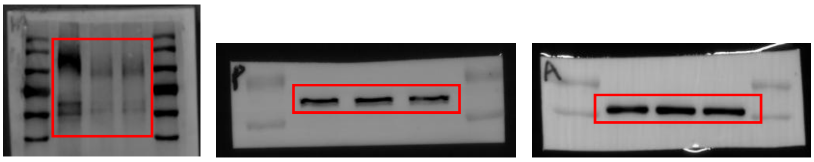

UMUC3

HA PTBP1 Actin

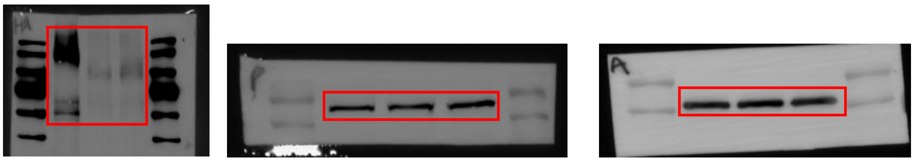

Figure 3B

T24

HA PTBP1 Actin

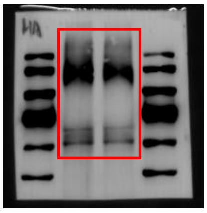 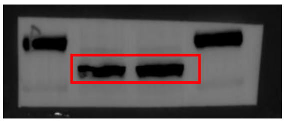 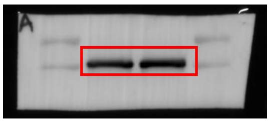

UMUC3

HA PTBP1 Actin

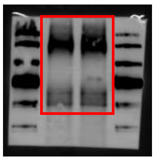 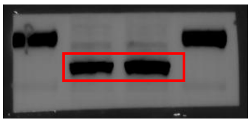 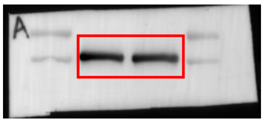

Figure 3C

T24

HA

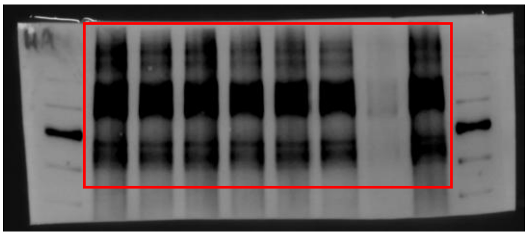

PTBP1 Actin

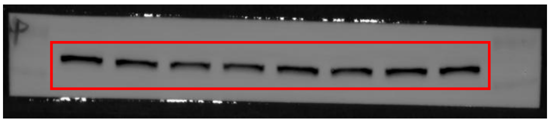 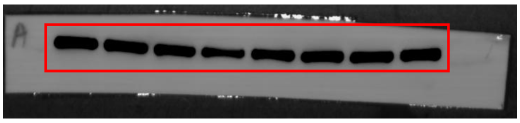

UMUC3

HA

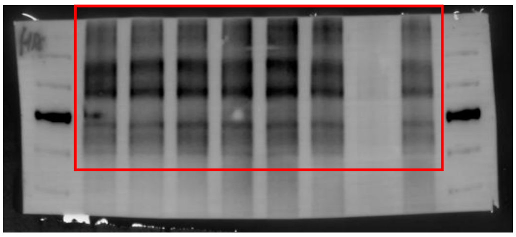

PTBP1 Actin

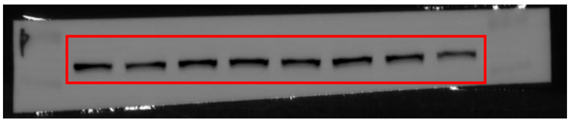 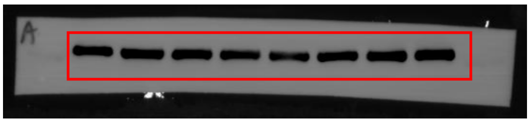

Figure 3D

T24

HA

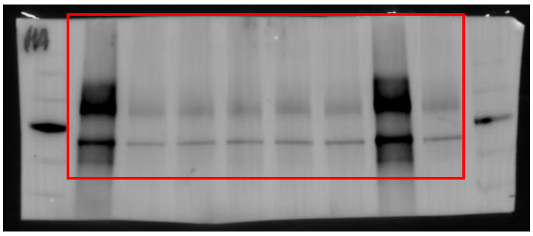

PTBP1

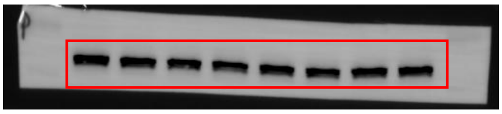

Actin

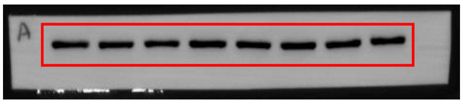

UMUC3

HA

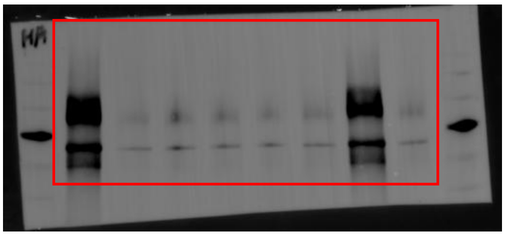

PTBP1

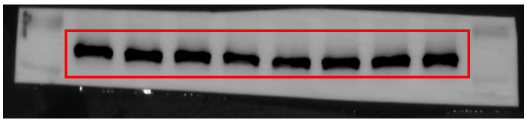

Actin

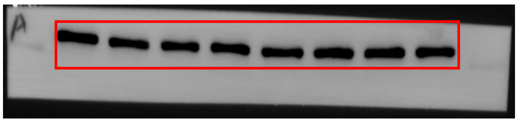

Figure 4D

CUL5

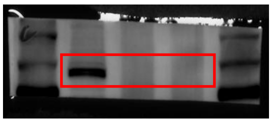

Actin

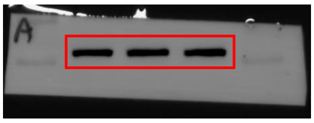

Figure 4E

PTBP1

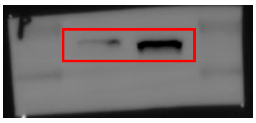

Actin

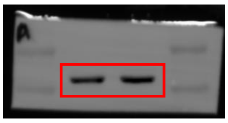

Figure 4F

PTBP1

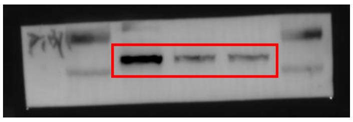

Actin

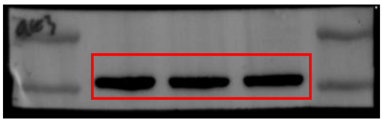

Figure 4G

CUL5

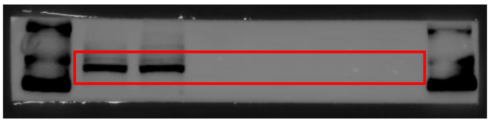

PTBP1

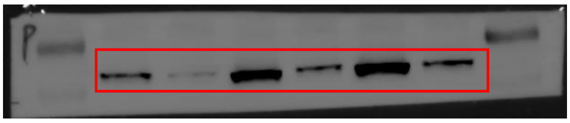

Actin

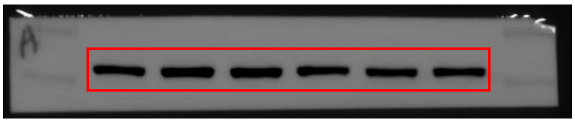

Figure 4H

CUL5

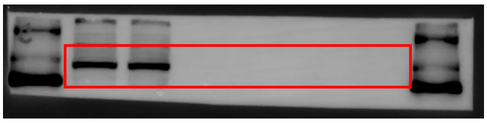

Actin

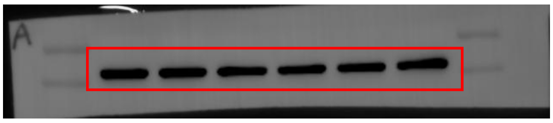

Figure 4I

PTBP1

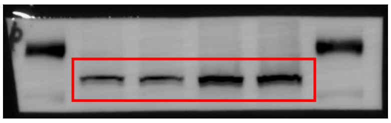

Actin

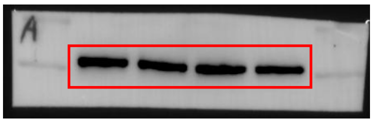

Figure 4J

PTBP1

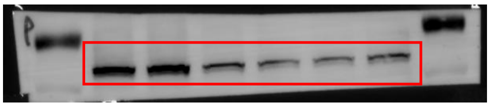

Actin

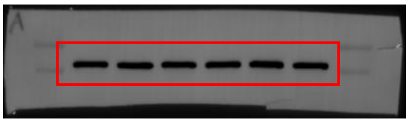

Figure 4K

PTBP1

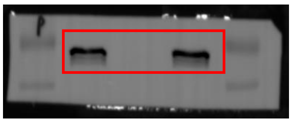

Figure 4L

Flag

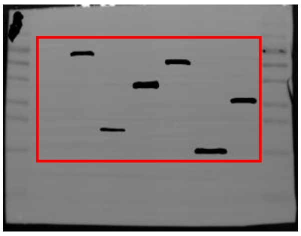

Actin

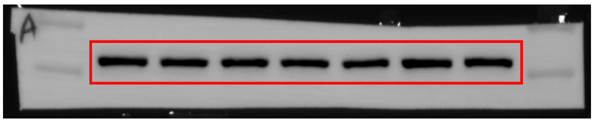

Figure 5A

Flag

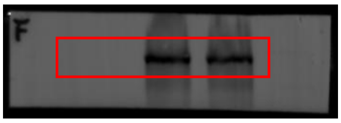

P62

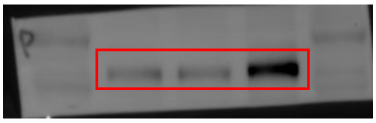

LC3

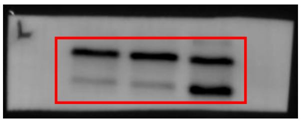

Actin

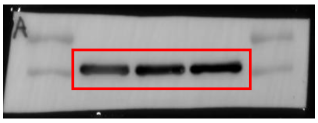

Figure 5D

P62

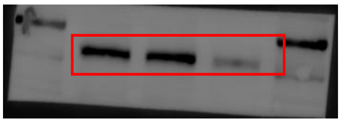

LC3

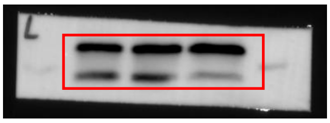

Actin

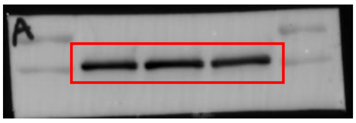

Figure 5E

Rubicon

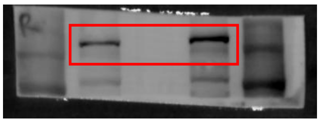

UVRAG

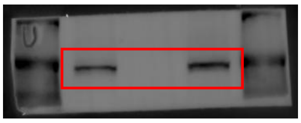

Beclin1

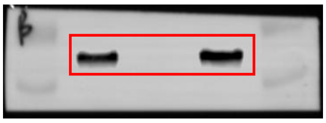

Figure 5F

Flag-Rubicon-L

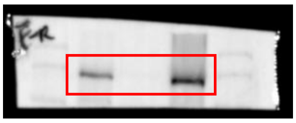

UVRAG

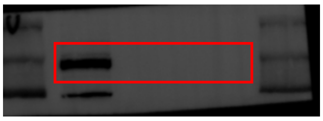

Beclin1

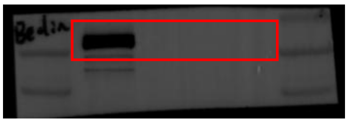

Flag-Rubicon-S

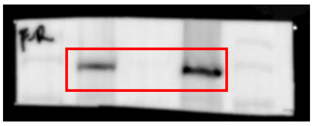

UVRAG

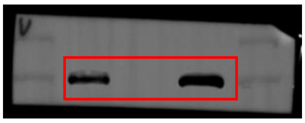

Beclin1

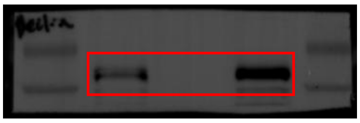

Figure 5G

CUL5

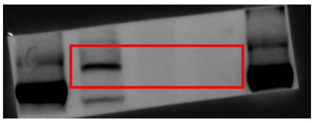

P62

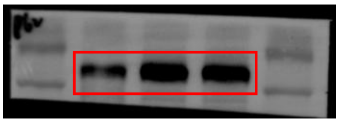

LC3

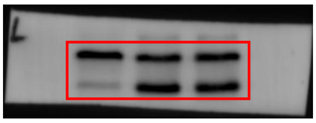

Actin

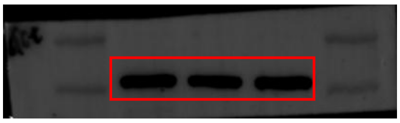

Figure 5I

CUL5

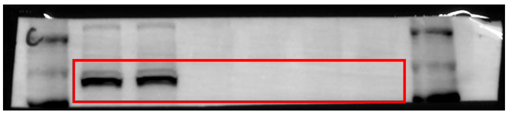

PTBP1

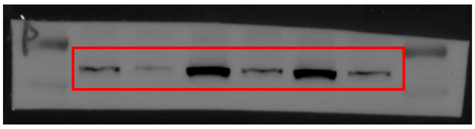

P62

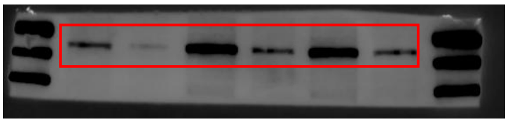

LC3

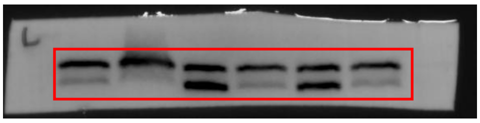

Actin

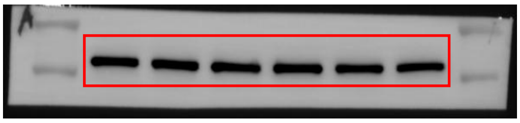

Figure 5J

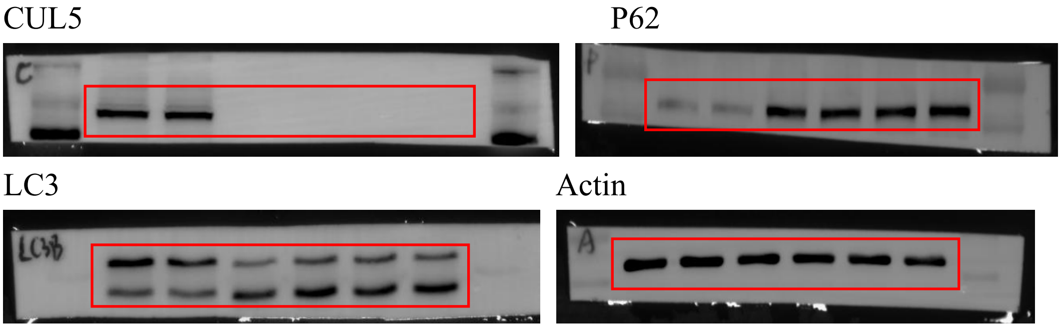

Figure 5L

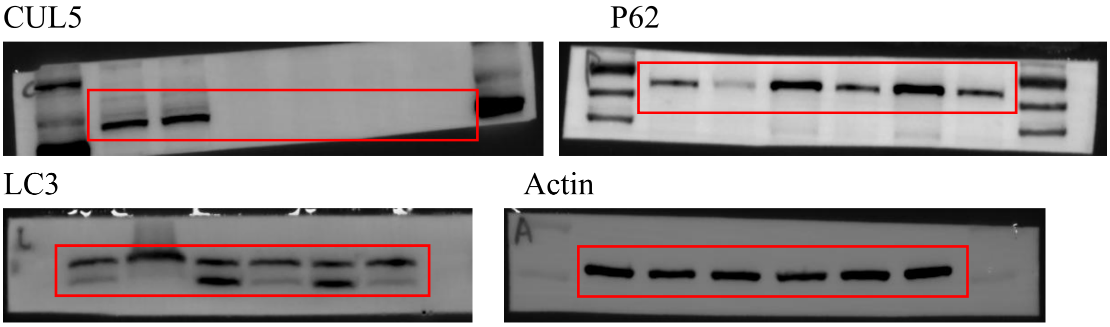

Figure 5N

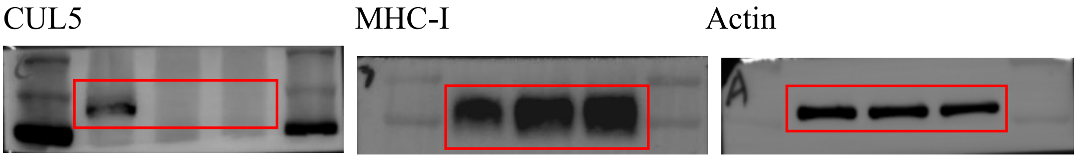

Figure S2A

PTBP1

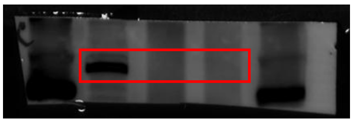

Actin

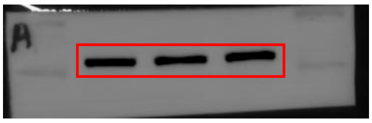

Figure S2B

PTBP1

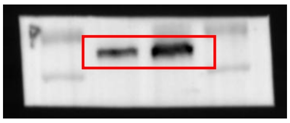

Actin

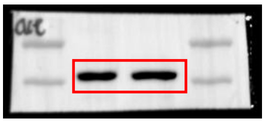

Figure S2C

PTBP1

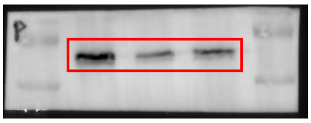

Actin

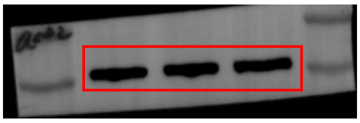

Figure S2D

CUL5

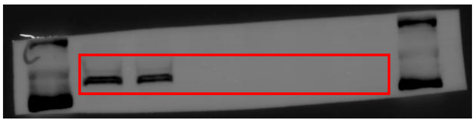

PTBP1

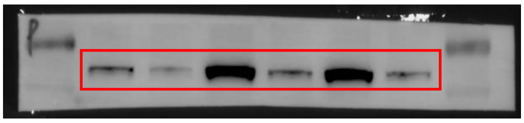

Actin

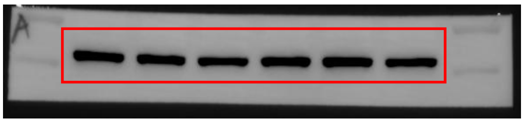

Figure S2E

CUL5

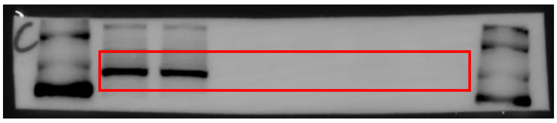

Actin

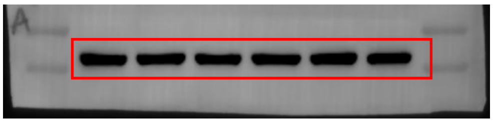

Figure S2F

PTBP1

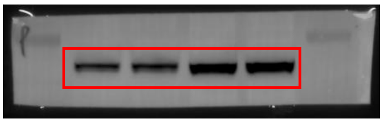

Actin

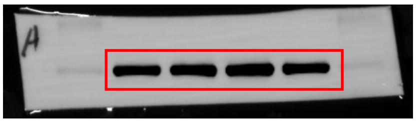

Figure S2G

PTBP1

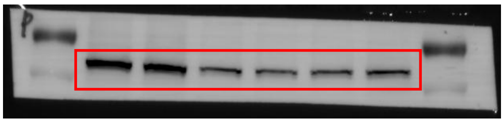

Actin

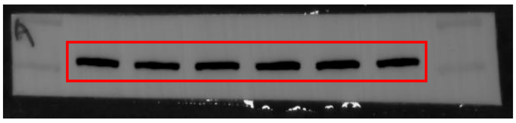

Figure S2H

PTBP1

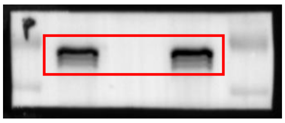

Figure S2I

Flag

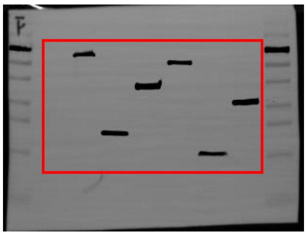

Actin

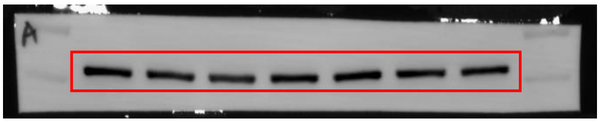

Figure S3A

Flag

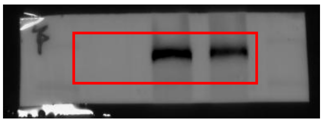

P62

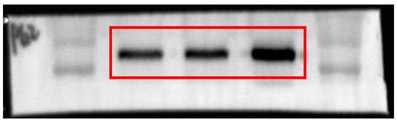

LC3

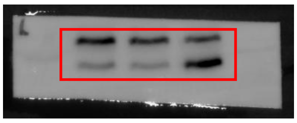

Actin

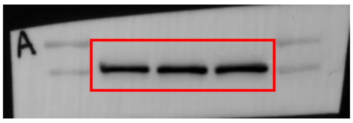

Figure S3D

P62

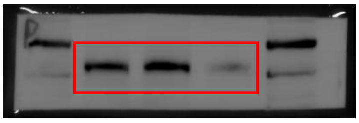

LC3

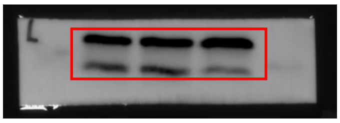

Actin

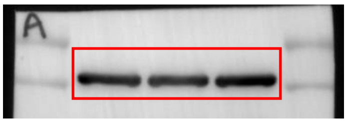

Figure S3E

Rubicon

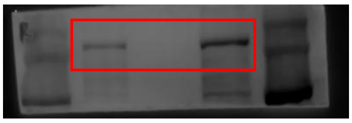

UVRAG

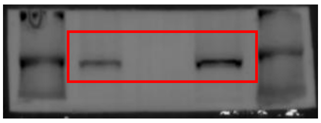

Beclin1

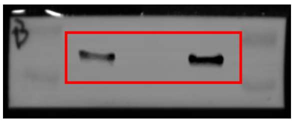

Figure S3F

Flag-Rubicon-L

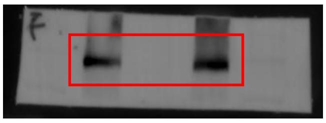

UVRAG

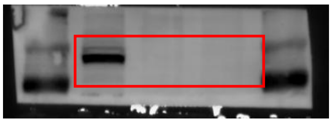

Beclin1

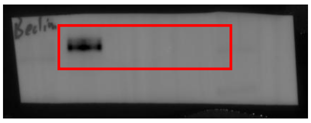

Flag-Rubicon-S

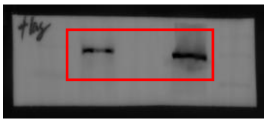

UVRAG

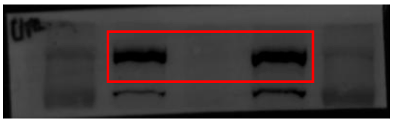

Beclin1

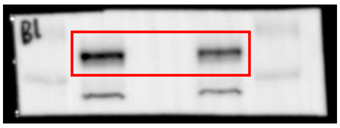

Figure S3G

CUL5

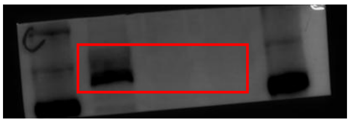

P62

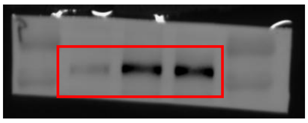

LC3

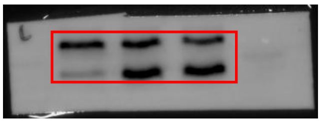

Actin

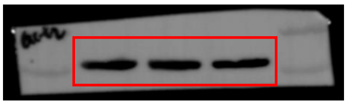

Figure S3I

CUL5

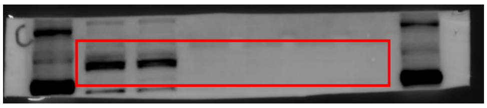

PTBP1

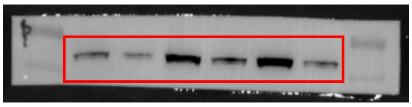

P62

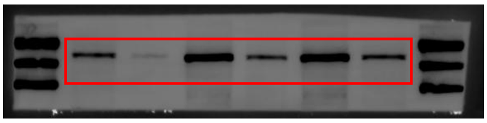

LC3

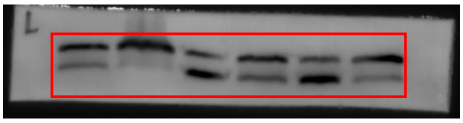

Actin

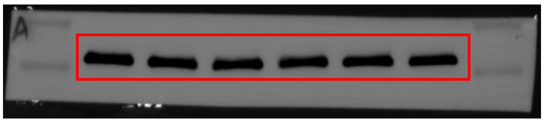

Figure S3J

CUL5

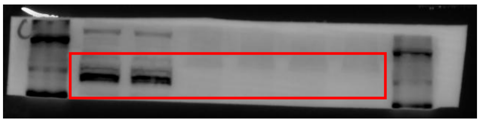

P62

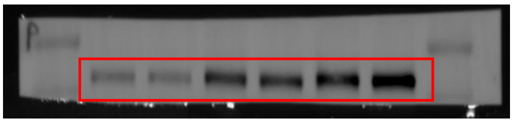

LC3

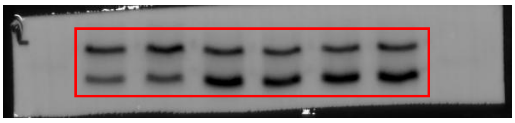

Actin

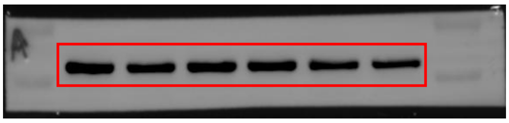

Figure S3L

CUL5

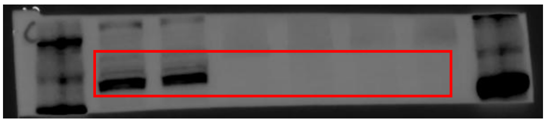

P62

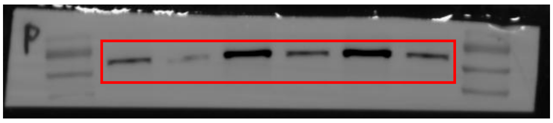

LC3

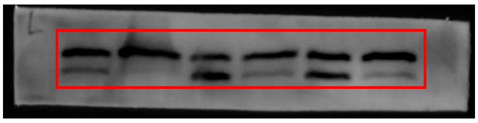

Actin

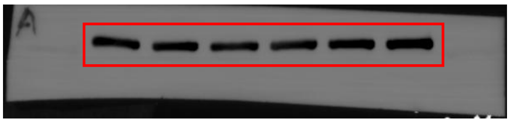

Figure S4A

A549

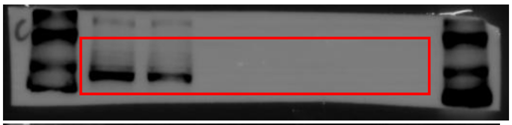

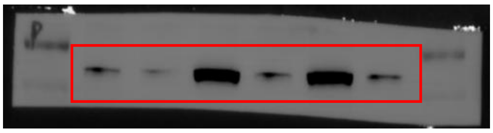

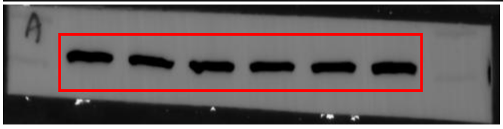

Figure S4B

A549

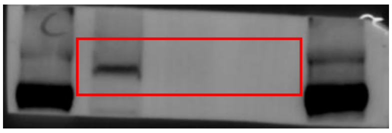

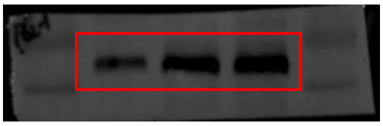

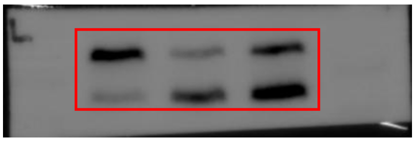

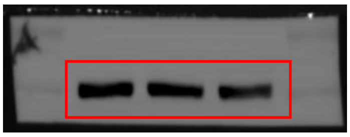

Original images of gels

Figure4D 、FigureS2A

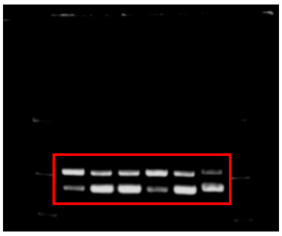

Figure4E、FigureS2B

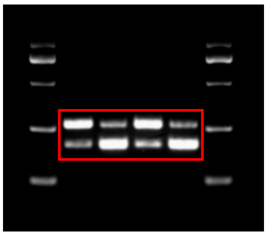

Figure4F、FigureS2C

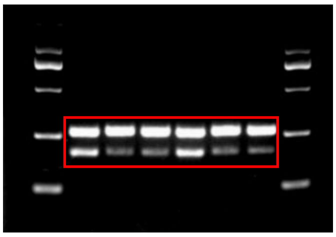

Figure4G

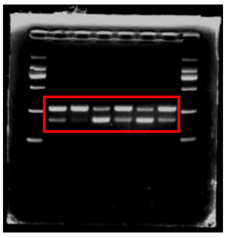

Figure4H

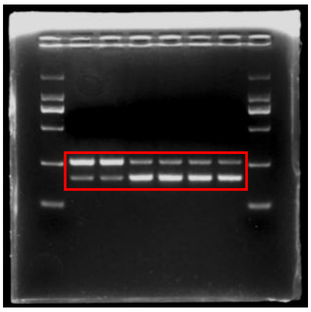

Figure4I

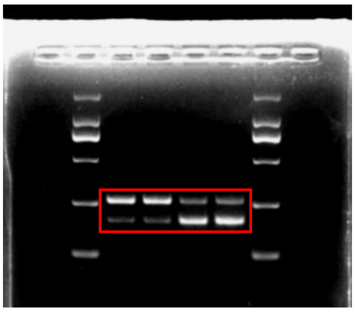

Figure4J

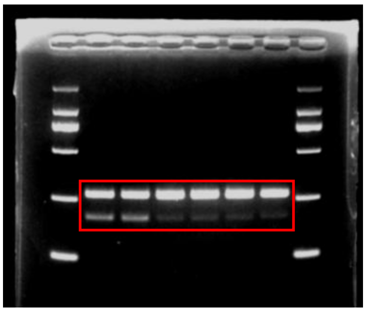

Figure4L

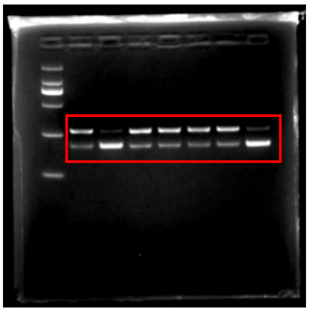

FigureS2D

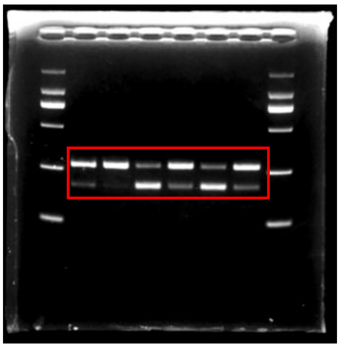

FigureS2E

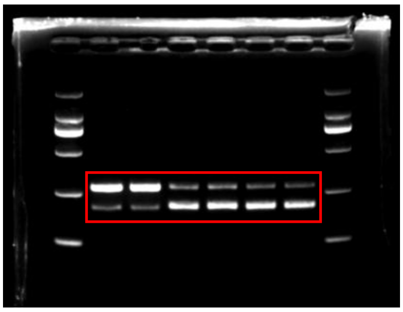

FigureS2F

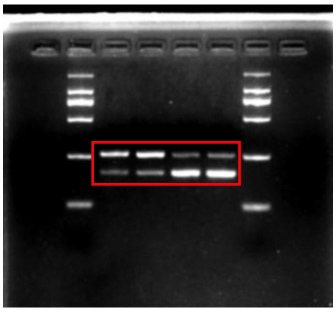

FigureS2G

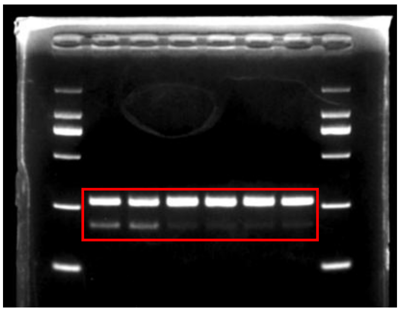

FigureS2I

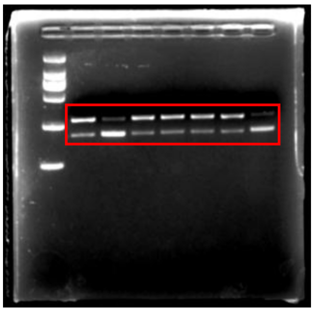

Figure5D

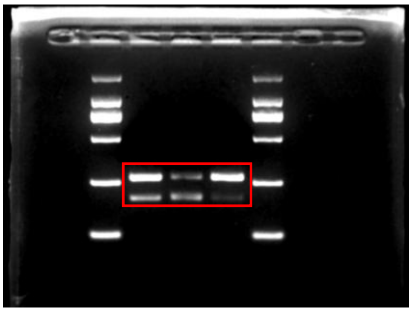

Figure5J

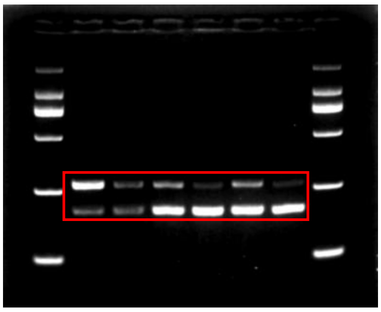

Figure5L

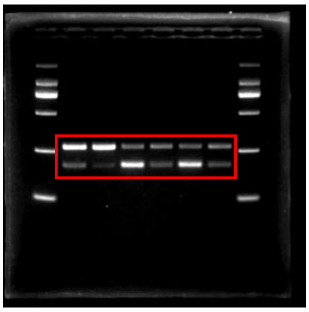

FigureS3D

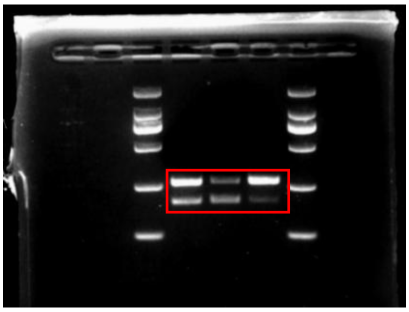

FigureS3J

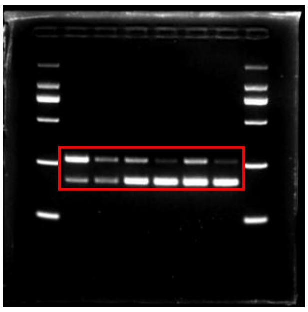

FigureS3L

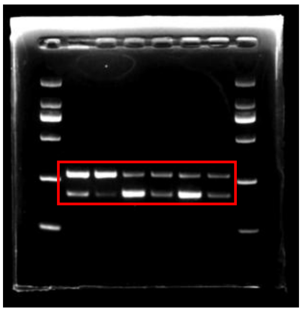

FigureS4A

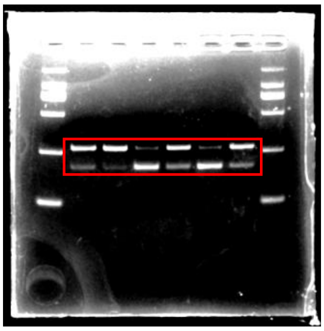

Supplement: S1 Raw Images — (PDF) [file pbio.3003647.s010.pdf]
